# Protein-responsive ribozyme switches in eukaryotic cells

**DOI:** 10.1093/nar/gku875

**Published:** 2014-10-01

**Authors:** Andrew B. Kennedy, James V. Vowles, Leo d'Espaux, Christina D. Smolke

**Affiliations:** 1 Department of Bioengineering, 443 Via Ortega, MC 4245 Stanford University, Stanford, CA 94305, USA; 2 Division of Chemistry and Chemical Engineering, 1200 E. California Boulevard, MC 210-41, California Institute of Technology, Pasadena, CA 91125, USA

## Abstract

Genetic devices that directly detect and respond to intracellular concentrations of proteins are important synthetic biology tools, supporting the design of biological systems that target, respond to or alter specific cellular states. Here, we develop ribozyme-based devices that respond to protein ligands in two eukaryotic hosts, yeast and mammalian cells, to regulate the expression of a gene of interest. Our devices allow for both gene-ON and gene-OFF response upon sensing the protein ligand. As part of our design process, we describe an *in vitro* characterization pipeline for prescreening device designs to identify promising candidates for *in vivo* testing. The *in vivo* gene-regulatory activities in the two types of eukaryotic cells correlate with *in vitro* cleavage activities determined at different physiologically relevant magnesium concentrations. Finally, localization studies with the ligand demonstrate that ribozyme switches respond to ligands present in the nucleus and/or cytoplasm, providing new insight into their mechanism of action. By extending the sensing capabilities of this important class of gene-regulatory device, our work supports the implementation of ribozyme-based devices in applications requiring the detection of protein biomarkers.

## INTRODUCTION

Proteins are the primary determinants of cellular phenotype, and much of cellular behavior is governed by protein concentrations and activities. Therefore, genetic devices that directly detect and respond to intracellular concentrations of proteins are important engineering tools. By linking protein concentrations to gene expression events, researchers can build synthetic gene control systems that target, respond to or alter specific cellular states.

Synthetic RNA switches are a class of genetic devices that regulate target gene expression in response to user-specified molecular inputs. They generally contain at least two core components: a sensor component (typically an aptamer that binds a small molecule or protein ligand) that detects the input signal through a binding interaction and an actuator component that modulates expression of the target gene. Many such binding elements can be found in nature (1–3) and new aptamers can be generated to target ligands through *in vitro* selection strategies (4,5).

A number of RNA devices that respond to protein ligands have been demonstrated in higher eukaryotes, including mammalian cells (6–10). These protein-responsive systems have been shown to act through various mechanisms, including translational inhibition (8,10–12), splicing regulation (13) and RNAi-based gene silencing (9). However, the protein-responsive RNA devices demonstrated to date exhibit a number of functional limitations. For example, based on the mechanism of gene regulation encoded in the switch, the gene-regulatory device can respond to protein ligands in the nucleus or the cytoplasm, but not both, which can limit the applications of these existing platforms. In addition, most of the device platforms described to date utilize an architecture in which ligand binding is linked to the modulation of the regulatory component's activity through a single mechanism, resulting in platforms that exhibit a single input/output (I/O) relationship (i.e. either ON or OFF but not both). Finally, the protein-responsive RNA devices described to date are not readily portable among higher eukaryotes, simpler microorganisms and *in vitro* systems, limiting the capability to perform rapid prototyping and device optimization strategies (14,15).

As an alternative RNA device platform, ligand-responsive ribozyme switches can regulate cleavage events in mRNAs to modulate the stability of the transcript in response to ligand levels. A previously described framework for constructing ribozyme-based devices provides a modular strategy for assembling this class of gene-regulatory devices from a sensor component, comprising an RNA aptamer, an actuator component, comprising the satellite RNA of tobacco ringspot virus (sTRSV) hammerhead ribozyme (HHRz) (16,17) and a transmitter component, comprising a sequence that functionally couples the sensor and actuator components (18). Ribozyme switches have been used in a variety of cellular engineering applications to date, including performing multi-input logic operations (19), supporting high-throughput enzyme evolution strategies (20) and controlling cell fate decisions (21,22).

The ribozyme switch platform addresses a number of the limitations associated with the protein-responsive RNA devices demonstrated to date. First, the transmitter component supports the rational design of ribozyme switches that either repress or enhance gene expression, allowing the platform to access both ON and OFF I/O relationships (15,18–19,21). Second, switch activity and function can be tuned through modifications to the sequence of the aptamer, ribozyme, and transmitter components (18,23). Third, because their mechanism of action is independent of cell-specific machinery, ribozyme switches exhibit conserved activity across higher eukaryotes, microorganisms and *in vitro* systems (14–15,18,23). However, ribozyme switches have only been demonstrated to respond to small molecule ligands. In addition, the precise mechanism of action of this class of RNA device (i.e. where cleavage takes place within the cell) and thus requirements for ligand localization for modulating cleavage activity is not currently understood.

Here, we demonstrate the extension of the ribozyme switch platform to the detection of protein ligands. We develop two device architectures that incorporate different structure switching mechanisms to control ribozyme cleavage activity as a function of ligand binding to the sensor domain of the device. We also demonstrate that an *in vitro* characterization pipeline can be used to prescreen device designs to identify the most promising candidates for *in vivo* testing and validation. Specifically, the *in vitro* screen can be used to identify protein-responsive ribozyme switches with gene-regulatory activities in both yeast and mammalian cells. We observe that *in vivo* gene-regulatory activities in the two types of eukaryote cells correlate with *in vitro* cleavage activities determined at different, physiologically relevant magnesium ion concentrations. Finally, localization studies with the protein ligand demonstrate that ribozyme switches can respond to ligands localized in the nucleus, the cytoplasm or both, providing new insight into the mechanism of action of this important class of gene-regulatory devices.

## MATERIALS AND METHODS

### Preparation of *cis*-blocked RNA devices and protein ligands for *in vitro* assays

*Cis*-blocked RNA devices transcribed from DNA templates were prepared as previously described (14). For *cis*-blocked ribozyme constructs (Supplementary Table S1), polymerase chain reaction (PCR) products were amplified from DNA templates using the forward primer T7-fwd (5′-TTCTAATACGACTCACTATAGG) and the reverse primer bMS2-rev (5′-AACAAAG CTGTTTCGTCCTC). His-tagged-maltose binding protein-MS2 coat protein (His-MS2-MBP), gift of Rachel Green (Johns Hopkins University), was used in all *in vitro* studies with MS2-responsive RNA devices.

### *In vitro* surface-plasmon-resonance-based RNA-protein binding assays

Binding data was determined for the protein–RNA device interaction by a surface-plasmon-resonance (SPR)-based assay, using a multi-cycle kinetics approach. Interactions were measured at several analyte concentrations as a function of time, and fit to a 1:1 binding model (24). The Biacore X100 (GE Healthcare Bio-Sciences, Uppsala, Sweden) sensor chip surface immobilized with DNA activator was generated as previously described (14). System priming, startup, and regeneration procedures were carried out as previously described (14), with HBS-EP+ (GE Healthcare Bio-Sciences) used as the running buffer.

Following the startup cycles, assay cycles were performed at several different analyte concentrations (0.1–600 nM), with at least one cycle repeated. Ligand capture amounts and analyte flow rates were varied in replicate binding assays for the same RNA device in effort to assess and minimize any mass transfer limitations (25,26). From first run dissociation constant (*K*_D_) approximations, the analyte concentrations of subsequent runs were adjusted, where necessary, such that they ranged from 0.1 × *K*_D_ to 100 × *K*_D_ for a more accurate estimate of the kinetic parameters (25,26).

Each assay cycle includes a capture, association, dissociation and regeneration step. The capture and regeneration steps were performed as described for the startup cycles. The association step was performed by an injection of HBS-EP+ buffer containing the protein analyte over both flow cells (FCs) for 30–90 s at 20–50 μl/min. Running buffer (HBS-EP+) was then injected over both FCs in the dissociation step for 300–600 s at 20–50 μl/min. The Biacore X100 Evaluation Software v2.0 (GE Healthcare Bio-Sciences) was used to process the datasets and analyze interaction kinetics. The reference flow cell (FC1) data was first subtracted from sample flow cell (FC2) to correct for injection noise, baseline drift, nonspecific surface binding and bulk refractive index changes. This reference-subtracted (FC2-FC1) data was further referenced to a blank injection of running buffer to account for any systematic drift occurring over course of the assay (24,27). The double-referenced data were fit to a 1:1 binding model for kinetic analysis (26). Reported values are the mean ± 1 standard deviation of at least three independent experiments.

### *In vitro* surface-plasmon-resonance-based RNA cleavage assays

SPR-based RNA cleavage assays were carried out for protein-responsive RNA devices as previously described for small molecule-responsive RNA devices (14). The processed sensorgram (*R*) was fit to a simple exponential equation *R* = (*R*_0_ − *R*_∞_) × (*e^−kd^*^t^) + *R*_∞_, where *R*_0_ (fit globally for a given replicate) is the initial SPR signal before the cleavage reaction, *R*_∞_ (fit locally for a given replicate) is the residual response at the end of the cleavage reaction and *kd* is the first-order RNA dissociation rate constant. RNA dissociation rate constants were only quantified in the absence of protein, as the SPR response signal from protein binding was significant, masking the RNA dissociation event response signal. Reported values are the mean ±1 standard deviation of at least three independent experiments.

### *In vitro* gel-based RNA cleavage assays

Generation of radiolabeled, full-length RNA devices responsive to protein ligands and subsequent cleavage assays to determine cleavage kinetics were carried out as previously described for natural HHRzs and theophylline-responsive RNA devices with minor adaptation (14). Briefly, gel-based ribozyme cleavage assays were performed in a physiologically relevant reaction buffer (40 μl) composed of 500 μM MgCl_2_, 150 mM NaCl, 1 mM DTT and 10 mM HEPES (pH 7.4) at 37°C. In the reaction volume, 1–10 nM of radiolabeled, full-length RNA generated from the *cis*-blocking strategy was first incubated with 2.5μM DNA activator strand (5′-AAACAACTTTGTTTGTTTCCCCC), for 5 min to activate the blocked RNA. The zero time-point aliquot was taken before initiating the self-cleavage reaction with the addition of MgCl_2_ and indicated amount of His-MS2-MBP fusion protein. To determine *k*_obs_, the first-order rate constant of *in vitro* RNA self-cleavage, the cleaved product fraction at each time point (*F*_t_) was fit to the single exponential equation *F*_t_ = *F*_0_ + (*F*_∞_ − *F*_0_) × (1 – *e*^−*k*obst^) using GraphPad Prism 5 (GraphPad Software, La Jolla, CA), where *F*_0_ and F_∞_ are the fractions cleaved before the start of the reaction and at the reaction endpoint, respectively.

For selected MS2-responsive RNA devices, additional cleavage assays were performed at various MS2 protein concentrations to generate dose-response curves. The cleavage rate constant (*k*_obs_) at each MS2 protein concentration ([MS2]) was fit to the sigmoidal equation *k*_obs_ = *k*_min_ + (*k*_max_ − *k*_min_) / (1 + [MS2] / IC_50_) using GraphPad Prism 5, where *k*_max_ and *k*_min_ are the maximum and minimum cleavage rate constants, evaluated in the absence of and with the highest MS2 concentration assayed, respectively (28–30). The IC_50_ is defined as the MS2 protein concentration at which the cleavage rate constant is half-maximal. In all regressions, the model fit the data well with *R*^2^ > 0.95.

### Plasmid construction

All plasmids were constructed using standard molecular biology techniques. Oligonucleotides were synthesized by Integrated DNA Technologies (Coralville, IA) and the Stanford Protein and Nucleic Acid Facility (Stanford, CA). Cloning enzymes, including restriction enzymes and T4 DNA ligase, were obtained from New England Biolabs and Life Technologies. Cloning products were electroporated into *Escherichia coli* DH10B (Life Technologies) using a GenePulser XP system (Bio-Rad Laboratories) or transformed into *E. coli* One Shot Top 10 (Life Technologies) using standard methods. Clones were screened using colony PCR and verified by sequencing (Elim Biopharmaceuticals, Hayward, CA).

The yeast MS2 expression vector (pCS2711; Supplementary Figure S1A) was based on plasmid 14191 (Addgene, Cambridge, MA), into which a DNA fragment encoding an MS2-mCherry fusion protein, obtained from pCS2580, was inserted between a TDH3 promoter and a CYC1 terminator by Gateway cloning methods (31). The plasmid 14192 (Addgene) was used as the no-MS2 empty control vector. All ribozyme-based devices and associated controls were cloned between a yeast-enhanced green fluorescent protein (GFP) open reading frame and ADH1 terminator in pCS1748 using either Gibson cloning or restriction enzyme-mediated cloning (AvrII/XhoI) as described previously (23) (Supplementary Figure S1B).

A standardized cloning method was developed to facilitate insertion of ligand-responsive devices and ligand coding regions into a single plasmid backbone that was compatible with the construction of stable isogenic mammalian cell lines. A DNA fragment encoding d2EGFP with a bGHpA signal and the cytomegalovirus (CMV)-TetO2 promoter was synthesized by GeneArt (Life Technologies) and inserted into pcDNA5/FRT (Life Technologies) between the restriction sites AflII/KpnI to form pCS2304 (Supplementary Figure S1C), which contains a CMV promoter expressing d2EGFP and FRT recombinase sites compatible with single-site, stable integration into the genome of Flp-In T-REx human embryonic kidney 293 (HEK293) cells (Life Technologies). A DNA fragment encoding the EF1α promoter upstream of the blue fluorescent protein (BFP) coding region was PCR-amplified from pCS2585 (courtesy Melina Mathur) using the primers EF1BFP Fwd and EF1BFP Rev and inserted between BglII/AvrII, and the MS2 coding region was PCR-amplified from pCS1392 using the primers No NLS A/X Fwd and MS2 A/X Rev and inserted between XhoI/ApaI in pCS2304 to form pCS2595 (Supplementary Figure S1D).

A DNA fragment encoding 2MS2mut (MS2 V75E/A81G head-to-tail fused dimer) was synthesized by GeneArt (Life Technologies) and inserted into pCS2595 between NotI/ApaI to form pCS2686. The coding region of 2MS2mut was PCR-amplified from pCS2686 using the primers NLS MS2 F and 2MS2mut R to add an N-terminal nuclear localization sequence (NLS), and using the primers 2MS2mut F and NES MS2 R to add a C-terminal nuclear exclusion signal (NES), and inserted into pCS2595 between NotI/ApaI to form pCS2747 and pCS2787, respectively.

The coding region of DsRedMonomer was PCR-amplified from pCS2359 using the primers DsRed GF and DsRed GR, and using the primers DsRed GF and DsRed NES R to add a C-terminal NES. The resulting DNA fragments were inserted into plasmids digested with ApaI using Gibson assembly (32) to create 2MS2mut-DsRedMonomer fusions as follows: DsRed into pCS2686 to form pCS2897, DsRed into pCS2747 to form pCS2902, and DsRed-NES into pCS2686 to form pCS2907. The ribozyme-based devices and associated controls (Supplementary Table S1) were inserted between the AvrII/AscI cloning sites in all plasmids (pCS2595, pCS2686, pCS2747, pCS2787, pCS2897, pCS2902, pCS2907).

### RNA device characterization assays in yeast

All yeast strains described in this work are based on CSY22, a previously-described W303a-derived strain with a *GAL2* deletion (33). Yeast transformations were performed using standard lithium acetate methods (34). Cultures were grown in synthetic complete media, 2% dextrose, YNB (Life Technologies), lacking uracil and tryptophan. Fluorescent protein expression levels were measured through flow cytometry analysis. Briefly, overnight cultures were backdiluted 1:100 into fresh 3-ml cultures and grown 6 h at 30°C. Fluorescence levels of cell populations were measured on a FACSAriaII Custom System (BD Biosciences, San Jose, CA) equipped with a 488-nm laser, with emission collected at 508 nm. Data were analyzed using FlowJo (Tree Star, Ashland, OR). For each culture, viable cells were gated by side scatter and forward scatter, and the geometric mean GFP level of the population was recorded. For each test condition, the population geometric means of three independent replicates were averaged. These average values were then normalized by the average GFP of cells harboring an inactive ribozyme control (sTRSVctrl) assayed under the same conditions. Reported values are normalized averages ± 1 standard deviation.

### Mammalian cell culture

Flp-In T-REx HEK293 cells (Life Technologies) were cultured in 10 ml (10-cm dish) or 3 ml (6-cm dish) Dulbecco's Modified Eagle's medium (DMEM) (Life Technologies) supplemented with 10% fetal bovine serum (FBS) (Life Technologies), 100 mg/l zeocin (Life Technologies) and 5 mg/l blasticidin (Life Technologies) in a humidified incubator at 37°C and 5% CO_2_. Cells were seeded at 2 × 10^4^ cells/ml and passaged regularly using 0.25% trypsin-ethylenediaminetetraacetic acid (EDTA) (Life Technologies), with media replaced every 48–72 h. Cells stably integrated with Flp-In constructs were cultured similarly, except the cell culture media were supplemented with 100 mg/l hygromycin B (Life Technologies) and no zeocin.

### Transient transfection of mammalian cell lines

Flp-In T-REx HEK293 cells were seeded at 1 × 10^5^ cells/ml in 500 μl (24-well plate), 10 ml (10-cm dish) or 400 μl (8-chambered coverglass) DMEM with 10% FBS. The cells were transfected 21–27 h (flow cytometry assay), 48 h (cellular fractionation and extraction) or 24 h (confocal microscopy) after seeding with one or two plasmids using FuGENE HD (Promega, Madison, WI) according to the manufacturer's instructions. DNA and FuGENE HD were incubated together in Opti-MEM in a 1:3:50 (g:l:l) ratio for ∼1 h. 500-μl samples received 500 ng of DNA, 10-ml samples received 10 μg of DNA and 400-μl samples received 400 ng of DNA.

### Stable mammalian cell line generation

Flp-In T-REx HEK293 cells were seeded at 1 × 10^5^ cells/ml in 2 ml (6-well plate) DMEM with 10% FBS. The cells were cotransfected with a pcDNA5/FRT-derived plasmid and pOG44 (Life Technologies) 24 h later in a 1:9 ratio using FuGENE HD (Promega) according to the manufacturer's instructions. DNA and FuGENE were incubated together in Opti-MEM in a 1:3:50 (g:l:l) ratio for ∼1 h, with 2-ml samples receiving 2 μg of DNA. The cells were resuspended 24 h after transfection using 0.25% trypsin-EDTA and DMEM with 10% FBS, and one quarter of the cells were used to seed 2 ml (6-well plate) DMEM with 10% FBS. The media were replaced with DMEM with 10% FBS, 200-mg/l hygromycin B and 5-mg/l blasticidin 24 h later. The media were replaced every 72–96 h until macroscopic colonies were visible, usually after 10–14 days. Colonies were pooled together with 0.25% trypsin-EDTA and passaged into DMEM with 10% FBS, 100 mg/l hygromycin B and 5 mg/l blasticidin.

### RNA device characterization assays in mammalian cell lines

Doxycycline was added to the growth media to derepress the CMV-TetO_2_ promoter 18–28 h after seeding (30–75 min after transfection if applicable). Fluorescence data were obtained by flow cytometry 66–76 h after seeding using the MACSQuant VYB equipped with 405-, 488- and 561-nm lasers (Miltenyi Biotec, Bergisch Gladbach, Germany). Viability was gated by side scatter and electronic volume, and viable cells were further gated for BFP expression (transient transfections only). DsRed and BFP fluorescence was measured through 615/20 and 450/50 nm band-pass filters, respectively. Data were analyzed using FlowJo (Tree Star). Reported values are geometric mean ± 1 standard deviation from at least two biological replicates. Relative BFP expression levels are reported as the geometric mean fluorescence values normalized to those from cells harboring an inactive ribozyme control (sTRSVctrl) assayed under the same conditions.

## RESULTS

### Rational design of protein-responsive ribozyme switch platforms

**Figure 1.**
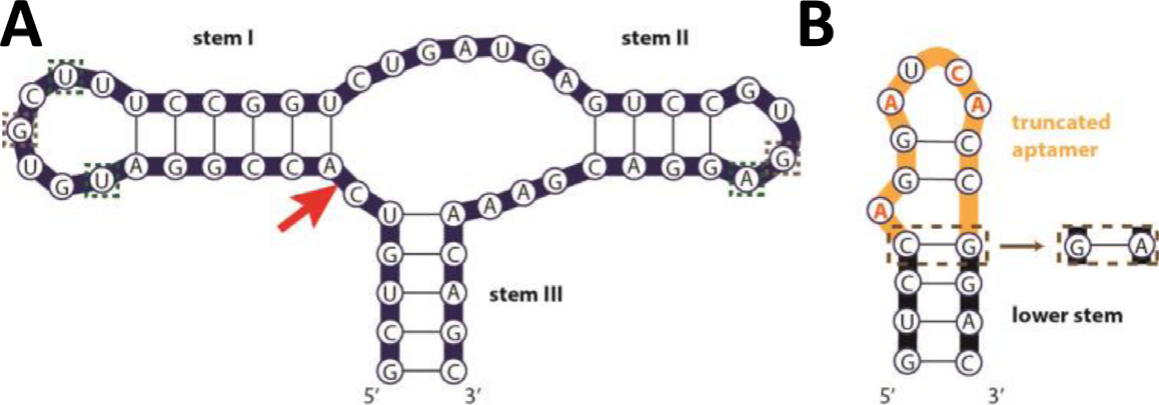
Sequence and secondary structures of the actuator (sTRSV HHRz) and sensor (MS2 aptamer) domains. (**A**) The sTRSV HHRz consists of a catalytic core flanked by three helices. The HHRz is integrated into the transcript through stem III and the loop sequences auxilliary to stems I and II are involved in mediating tertiary contacts required for catalytic activity at physiological Mg^2+^ concentrations. The Hoogsteen U-A-U base triple and GG base pair HHRz tertiary interactions (40) are denoted with boxed green and gray nucleotides, respectively. The site-specific phosphodiester bond isomerization that results in cleavage of the RNA is marked with a red arrow. (**B**) The high affinity variant of the MS2 bacteriophage translational operator RNA (38) used as the MS2 aptamer for all devices in this study. Bases involved directly in MS2 protein binding are indicated in orange, while all other bases are only structurally conserved (35). The MS2 F5 aptamer variant has a non-canonical GA pair (brown dotted boxes) below the bulged adenine (36). In device construction, the aptamer is truncated to the subsequence above the bulged adenine (yellow backbone) and grafted onto functional sequences (e.g. transmitter, HHRz stem) to reconstitute the aptamer lower stem (black backbone). All secondary structures were predicted by RNAstructure folding software (41).

We investigated different RNA device architectures for physically and functionally coupling the sTRSV HHRz actuator (Figure 1A) and MS2 coat protein aptamer sensor (Figure 1B) domains. The MS2 coat protein (35–38) was selected as a ligand input because it has a well-characterized binding interaction with RNA aptamers and due to its relatively small size is expected to be present in both the nucleus and the cytoplasm (39). Our designs leverage the fact that the lower stem of the aptamer (Figure 1B; black backbone bases) is only structurally conserved (35), which allows sequence substitutions in this region without a loss in binding affinity.

**Figure 2.**
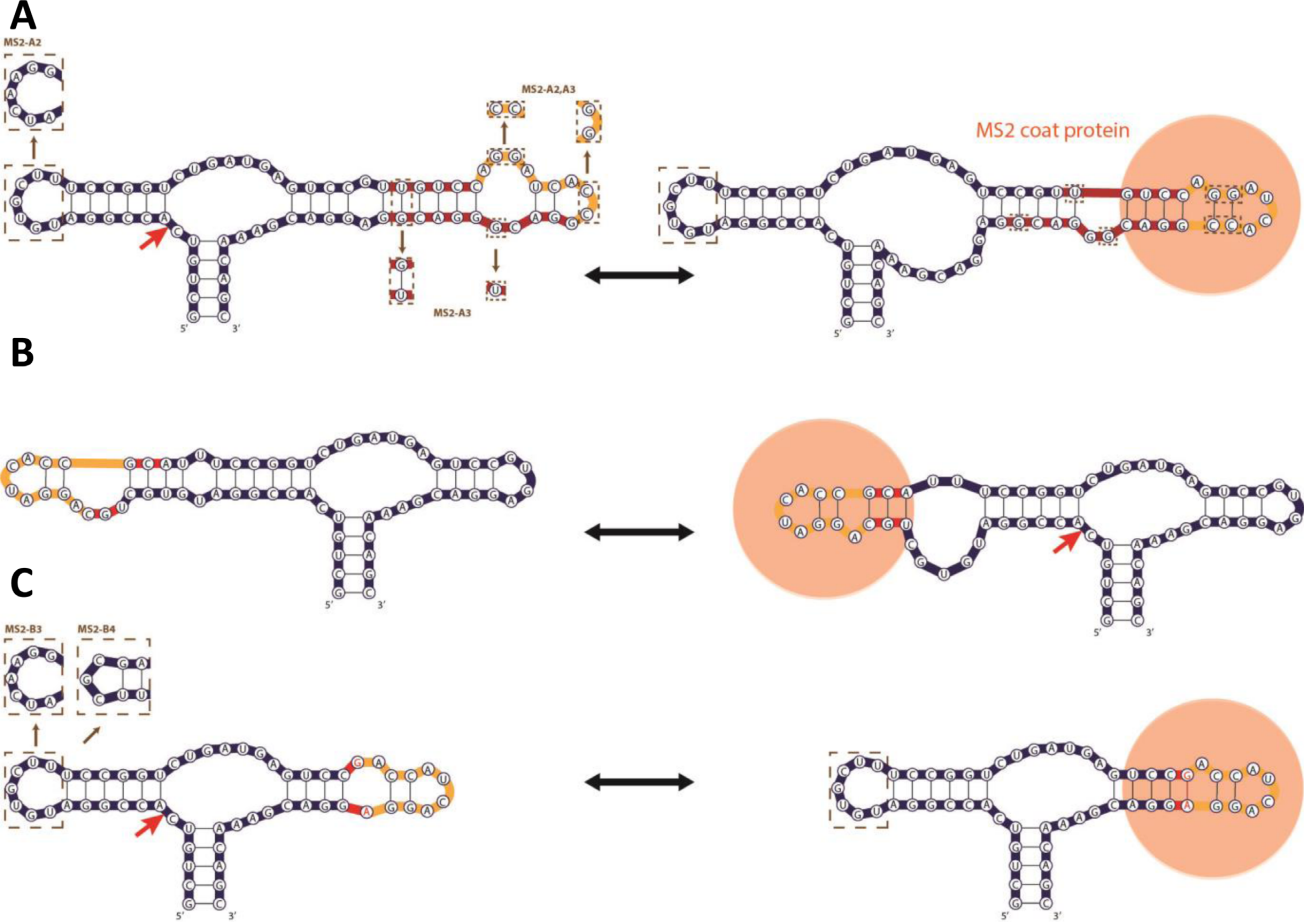
Structure switching designs of MS2-responsive RNA devices. Nucleotides that comprise the aptamer sensor, HHRz actuator and transmitter components are colored yellow, blue and red, respectively. In each panel the structure on the left is the MFE structure predicted by RNAstructure. (**A**) MS2-responsive transmitter-based RNA devices that utilize a secondary structure changing mechanism directed to disrupting the HHRz core (MS2-A). Predicted secondary structures of the active HHRz conformation with unformed MS2 aptamer (gene-OFF state, left) and the inactive HHRz conformation with formed MS2 aptamer (gene-ON state, right), bound with MS2 coat protein (orange) are shown. The sequence for the MS2-A1 RNA device is shown; brown dotted boxes denote sequence variations of MS2-A2 and MS2-A3 RNA devices, differing primarily in HHRz loop I and transmitter sequences, respectively. (**B**) MS2-responsive transmitter-based OFF switch RNA device with HHRz loop secondary structure changing mechanism (MS2-B1). In the MFE secondary structure both HHRz and aptamer are not formed (gene-ON state, left). In the active conformation both HHRz and aptamer are formed, shown bound with MS2 coat protein (gene-OFF state, right). (**C**) MS2-responsive transmitter-based ON switch RNA devices with HHRz loop secondary structure changing mechanism (MS2-B2, -B3, -B4). The HHRz structure is formed, but aptamer structure is not in the MFE structure (gene-OFF state, left). In the inactive ribozyme conformation, aptamer is formed and bound, and HHRz structure is disrupted (gene-ON state, right). These devices incorporate the MS2 aptamer variant containing a noncanonical GA pair (red text). The sequence for the MS2-B2 device is shown, brown dotted boxes denote sequence variations of the MS2-B3 and MS2-B4 devices, differeing in their HHRz loop I sequence.

In our first device architecture, the actuator and sensor domains are coupled through a separate transmitter component that mediates secondary structure switching promoted by ligand binding and directed to disrupting the HHRz core as previously described for small molecule-responsive RNA devices (18). MS2-responsive ON ribozyme switches, which up-regulated gene expression in the presence of ligand, were built with this core-disrupting transmitter architecture. Specifically, we modified a previously described theophylline-responsive ribozyme switch (L2b8 (18)) by replacing the theophylline aptamer with the truncated MS2 aptamer to create MS2-A1 (Figure 2A). We built a second MS2-responsive ribozyme switch, MS2-A2, by altering the loop I sequence of MS2-A1 to one which had been previously generated through a library screen and shown to result in higher ribozyme cleavage activities (23). We made a third transmitter-based device, MS2-A3, by flipping the base pair of the transmitter domain immediately proximal to the HHRz actuator to examine the role of this transmitter base pair (a GU wobble) in cleavage and switching activity. As the sequence of the upper stem of the MS2 aptamer (Figure 1B) is variable, two GC base pairs in this region were flipped in MS2-A2 and -A3 to avoid predicted (41) misfolding of the RNA device (Figure 2A). Four additional device designs (MS2-A4 to -A7) were made by varying the transmitter sequence in MS2-A1 (Supplementary Table S1) through sequential single base pair substitutions to examine the impact of varying the minimum free energies (MFEs) associated with the gene-ON and gene-OFF conformations (Supplementary Table S2). Two final device designs (MS2-A8, MS2-A9) were made by shortening the transmitter in MS2-A1 by two and four base pairs, respectively, and introducing base pair substitutions to vary the MFEs associated with each state.

The second device architecture is based on the incorporation of a transmitter that directs displacement of the HHRz loop structure (Figure 2B). As the HHRz loop sequences are involved in catalytically mediating tertiary interactions (42), we hypothesized that transmitter designs that modulated loop base pairing can also control cleavage activity of the RNA device. Both MS2-responsive ON and OFF ribozyme switches were designed with this loop-disrupting transmitter architecture. We created an OFF switch design, MS2-B1, by integrating the truncated MS2 aptamer with a three base pair transmitter into loop I (Figure 2B). The HHRz loop I integration point was selected based on control experiments indicating that this integration point retained high *in vitro* cleavage activity (data not shown). The transmitter is designed to Watson–Crick base pair with loop I bases in the MFE structure, thereby disrupting the cleavage-mediating loop tertiary interactions (Figure 2B). MS2 binding stabilizes a suboptimal structure in which the transmitter sequence base pairs to form the stem of the aptamer, freeing the loop I nucleotides to participate in cleavage-mediating tertiary interactions. In contrast, the ON switch loop-displacing designs incorporate a single base pair transmitter and variant MS2 aptamer to modulate HHRz loop II structure. The MS2 F5 aptamer (*K*_D_ ∼2 nM) was used in the design of these switches, which has a GC base pair in the stem mutated to GA (Figure 1B). The crystal structure of the F5 aptamer bound with its ligand shows that the GA nucleotides form a non-canonical base pair (37). We used this information to create an ON switch design, MS2-B2, by coupling the F5 aptamer sequence up to the GA base pair directly to stem II of the sTRSV HHRz, such that the GA of the aptamer replaced L2.1G and L2.4A of loop II (Figure 2C). We hypothesized that MS2 protein binding will cause the GA non-canonical pair to form, thereby closing loop II and inhibiting cleavage activity (Figure 2C). We built two additional ON switch designs, MS2-B3 and -B4, by altering the loop I sequence of MS2-B2 to non-natural sequences that had been previously shown to result in higher cleavage activities (23).

### *In vitro* characterization assays support a rapid screen for identifying candidate protein-responsive ribozyme switches for *in vivo* testing

**Figure 3.**
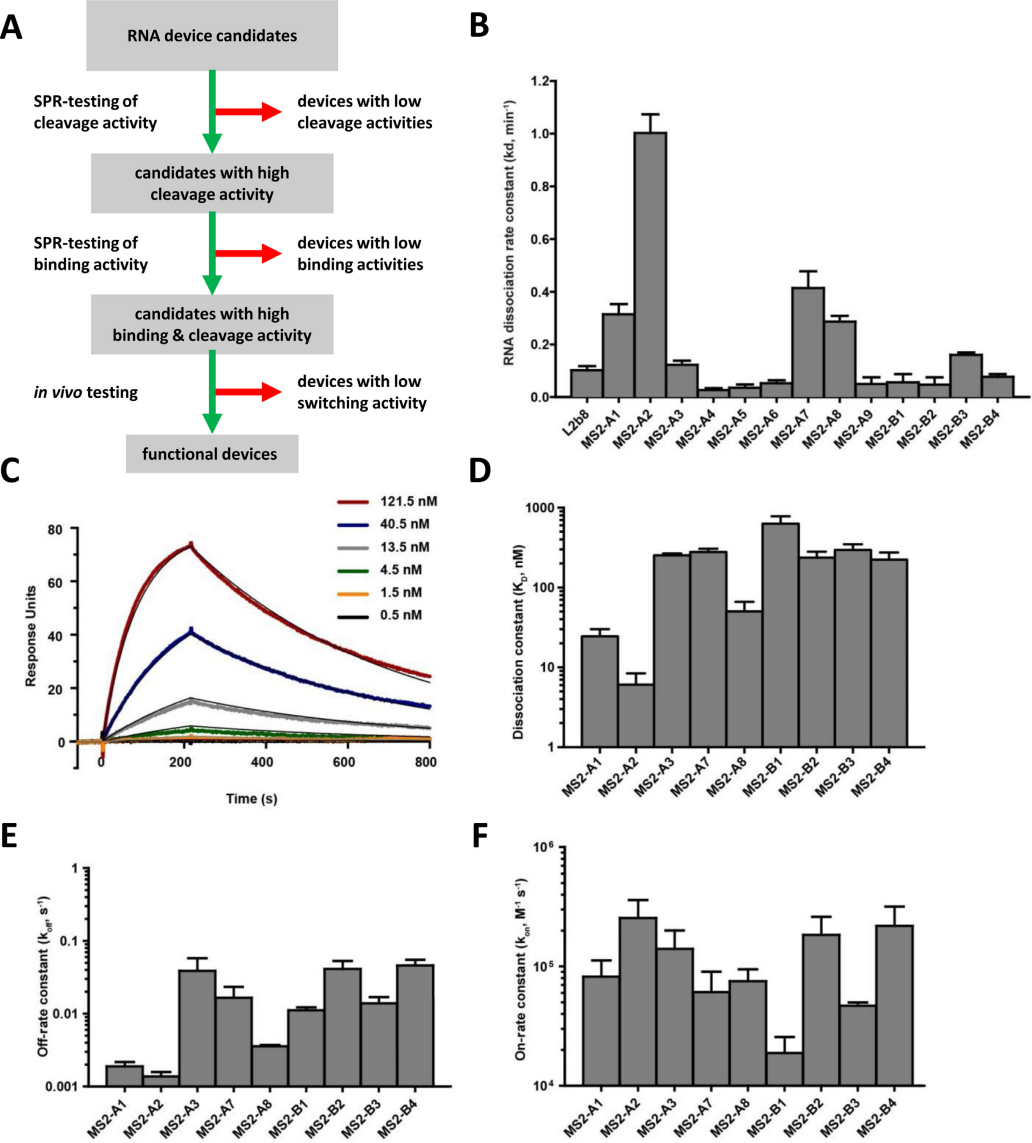
*In vitro* screening of protein-responsive RNA device cleavage and binding activities. (**A**) Process flowchart for development of protein-responsive RNA devices. Candidate RNA devices are first screened *in vitro* by rapid SPR-based assays for RNA cleavage activity, and then for ligand binding activity before *in vivo* testing. (**B**) SPR-based RNA dissociation rate constants for MS2-A and MS2-B RNA devices. Reactions were performed at 25°C, in 500 μM MgCl_2_, 150 mM NaCl and 10 mM HEPES (pH 7.4). Reported values are the mean ± SD of at least three independent assays. RNA device dissociation constants determined in absence of MgCl_2_ were all <0.01 min^−1^. (**C**–**F**) MS2 protein binding characterization of MS2-A and MS2-B RNA devices. (C) Representative SPR sensorgrams for the MS2-B1 RNA device. Overlaid in the plot is the association and dissociation portion of the sensorgrams from MS2 protein at six different concentrations (121.5, 40.5, 13.5, 4.5, 1.5, 0.5 nM). Representative sensorgrams for other RNA devices are presented in Supplementary Figure S3. Dissociation constants (*K*_D_; (D)) for each RNA device are fit by a 1:1 kinetic binding model, taking the ratio of the off-rate constant (*k*_off_; (E)) and on-rate constant (*k*_on_; (F)). Reported are the mean ± SD from at least three independent assays.

As part of the design process, numerous design variants are developed for each device architecture to increase the likelihood of arriving at a device design that exhibits desired gene-regulatory properties *in vivo*. Given the time associated with cloning and characterizing individual variants, a rapid way to prescreen designs for the most promising candidates is desirable. Recent studies have demonstrated that *in vitro* measured parameters, such as cleavage rates, can be good predictors of ribozyme switch function *in vivo* (14). Thus, we sought to develop and validate a set of *in vitro* assays for rapid characterization of key device parameters that can be used to prescreen ribozyme-based device designs prior to testing *in vivo* (Figure 3A).

To measure cleavage kinetics of the ribozyme-based devices, we leveraged a recently developed SPR-based assay for quantification of cleavage rate constants (14). In this method, RNA devices are captured onto the sensor surface, the cleavage reaction is initiated by addition of magnesium ions (Mg^2+^), and separation of the 3′ cleavage product from the sensor surface is monitored with decreasing SPR signal (Supplementary Figure S2). The kinetics of the SPR signal decrease during the cleavage reaction are fit to a one phase exponential decay equation. The fit RNA dissociation rate constant (*k*_d_) is reflective of the RNA cleavage rate constant (14). We applied the SPR-based cleavage assay to measure the RNA dissociation rate constants (*k*_d_) of the MS2-A and -B series devices in the absence and presence of 500 μM MgCl_2_. A previously characterized device, L2b8, was run as a benchmark to set a cutoff for identifying devices with cleavage activities expected to result in desired *in vivo* gene-regulatory activities. RNA dissociation rate constants determined in the absence of MgCl_2_ were all <0.01 min^−1^ (data not shown), indicating that the devices exhibit insignificant cleavage activity levels in the absence of Mg^2+^ (Figure 3B). The *k*_d_ values in the presence of MgCl_2_ ranged from near background (0.03 min^−1^, MS2-A4) to ∼10× higher than the benchmark control (1.0 min^−1^, MS2-A2) (Figure 3B). The devices MS2-A1, -A2, -A3, -A7, -A8 and -B3 exhibit cleavage activities equal to or greater than the benchmark control and thus were subjected to additional *in vitro* characterization of binding properties. We chose three additional devices (MS2-B1, -B2 and -B4) to carry forward into binding characterization, including the OFF switch, which exhibited a relatively low *k*_d_ (0.06 min^−1^), and the devices with the MS2 F5 aptamer variant.

We characterized the binding properties of the selected MS2-A and -B devices in a recently developed SPR-based binding assay (43). Protein binding and dissociation to RNA devices captured onto the sensor surface are monitored in real time as an increase and decrease in SPR signal, respectively. The kinetics are fit to a 1:1 binding model to calculate the protein binding on- and off-rate constants, *k*_on_ and *k*_off_, respectively. The dissociation constant (*K*_D_) is calculated from ratios of *k*_off_ and *k*_on_ assayed at several ligand concentrations (24) (Figure 3C, Supplementary Figure S3) (26). The MS2-A and -B device dissociation constants span nearly three orders of magnitude from ∼6 to ∼600 nM (Figure 3D, Supplementary Figure S3) and provide insight into the mapping of performance properties on device designs. For example, the relatively high *K*_D_, and low *k*_on_ of the MS2-B1 device (Figure 3D and F) may be due to the shorter, three base-pair lower stem of the aptamer within this device (Figure 2B) allowing fewer protein-binding contacts (35,44–45), compared to the four base-pair stem found in the other devices (Figure 2A and C) (44,46). The MS2-B2, -B3 and -B4 devices contain the MS2 F5 aptamer variant and differ by their loop I sequence. While their measured dissociation constants are similar (250 ± 50 nM) (Figure 3D), the MS2-B3 device exhibits lower *k*_off_ and *k*_on_ rates (Figure 3E and F). The data indicate that the MS2-B2 and -B4 devices have aptamer-formed structures with greater stabilities than the MS2-B3 device (Supplementary Table S2).

The SPR-based assay cannot be used to measure the impact of ligand binding on cleavage activity by quantifying the device cleavage activity in the presence of protein ligand, as the signal change due to protein association/dissociation and RNA dissociation are not easily separable. Therefore, as a proxy for device sensitivity to ligand, we used the *K*_D_ to characterize device binding in comparison to *K*_D_ of aptamers that have been successfully utilized within *in vivo* functioning RNA devices. All the characterized MS2-A and -B devices exhibit greater ligand affinities (Figure 3D; *K*_D_< 1 μM) than previously developed ligand-responsive RNA devices (18,47). Thus, based on the *in vitro* screen the MS2-A1, -A2, -A3, -A7, -A8, -B1 and -B3 devices are identified as good candidates for subsequent *in vivo* testing.

### Protein-responsive ribozyme switches exhibit ON and OFF activities in yeast cells

**Figure 4.**
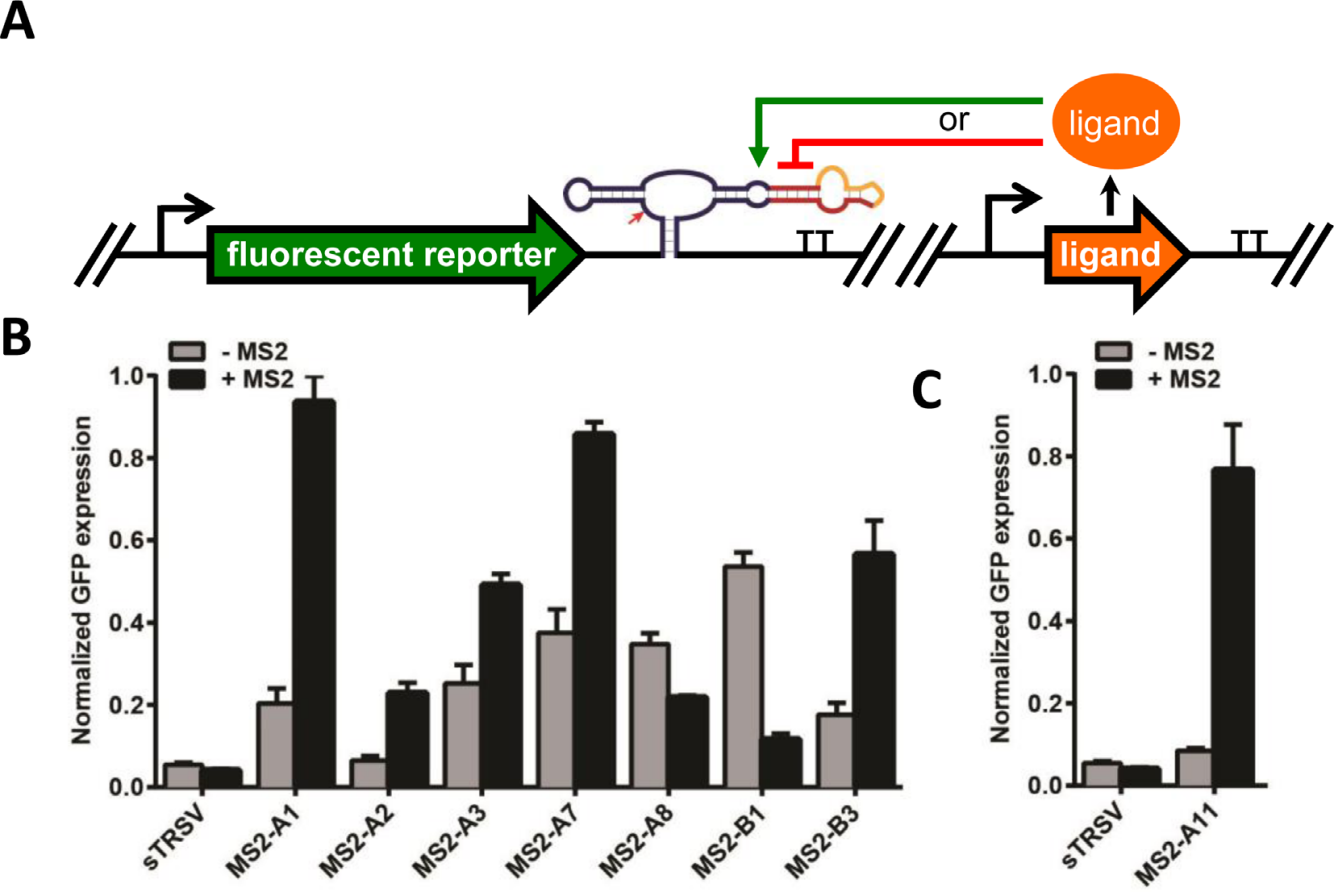
*In vivo* activity of protein-responsive ribozyme switches in yeast cells. (**A**) Schematic of the protein-responsive ribozyme switch characterization system for yeast. An expression construct encoding the ribozyme switch in the 3′ UTR of a fluorescent reporter (GFP) is encoded on a low-copy plasmid. A separate low-copy plasmid encodes the expression of the protein ligand (or an empty control). (**B**) Gene-regulatory activity of MS2-responsive ribozyme switches in yeast. (**C**) Gene-regulatory activity of optimized ribozyme switch (MS2-A11) in yeast. Relative GFP levels are reported for cells harboring the indicated ribozyme switch constructs or controls in the absence and presence of MS2. Reported values are the geometric mean ± SD from biological triplicates and normalized to the non-cleaving control (sTRSVctrl).

We characterized the *in vivo* activity of the ribozyme-based devices identified in the *in vitro* screen in a yeast host. Gene-regulatory activities of the ribozyme switches were characterized by placing the devices and associated controls in the 3′ untranslated region (UTR) of a fluorescent reporter protein (GFP) (Figure 4A). Device activities were measured as the mean fluorescent levels of cells harboring the indicated constructs in the absence and presence of MS2. The high-protein ligand condition was achieved by transforming a MS2 expression construct, encoding the expression of the MS2 coat protein fused to a mCherry protein, into the yeast cells. The no-protein ligand condition was achieved by transforming an empty plasmid, not encoding MS2 expression, into the yeast cells.

Ribozyme switch activities were characterized in yeast through flow cytometry analysis. An inactive HHRz control (sTRSVctrl) exhibited high GFP levels in the presence and absence of MS2, which we used to set the maximum GFP level. A wild-type HHRz (sTRSV) reduced gene expression to <5% of that observed from the inactive HHRz control, consistent with efficient ribozyme cleavage *in vivo*. The subset of MS2-responsive ribozyme switches tested in yeast exhibited significant changes in GFP levels in the presence and absence of MS2 (Figure 4B). The devices spanned a wide range of expression levels, and exhibited both ON- and OFF-switch activities. The ON switches exhibited a range of stringencies (basal expression levels in the absence of MS2) and exhibited activation ratios (ratio of expression in the presence and absence of MS2) up to 4-fold for ON switch MS2-A1, which switched from 20 to 97% of the maximal GFP level. The OFF switch MS2-B1 exhibited a 4.1-fold decrease in expression in the presence of MS2, spanning a range between 55 and 13% of maximal GFP levels. Non-switching control devices did not exhibit gene knockdown activity or responsiveness to MS2 under identical assay conditions (Supplementary Figure S4A), indicating that the observed gene-regulatory activity is due to ribozyme cleavage and modulation of cleavage through ligand binding rather than nonspecific effects. These results indicate that the *in vitro* screen was effective at identifying switches with *in vivo* gene-regulatory activities.

### Protein-responsive ribozyme switches exhibit ON and OFF activities in mammalian cells

**Figure 5.**
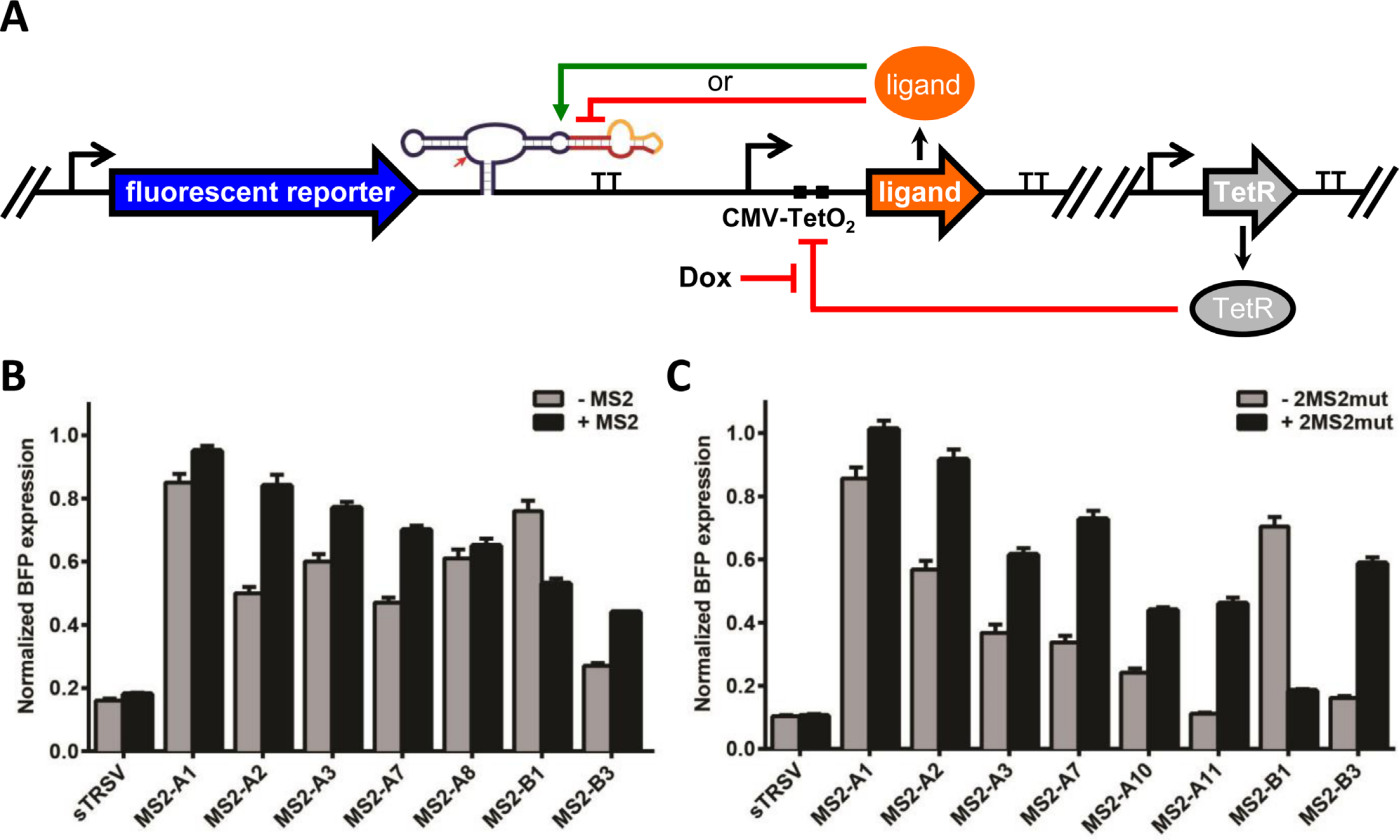
*In vivo* activity of protein-responsive ribozyme switches in HEK293 cells. (**A**) Schematic of the protein-responsive ribozyme switch characterization system for human cells. The first construct encodes the expression of the ribozyme switch in the 3′ UTR of a fluorescent reporter (BFP) from a constitutive promoter. The second construct encodes the expression of the protein ligand from a tetracycline-responsive CMV-TetO_2_ promoter. Both expression constructs are placed on a single plasmid, which is transfected into a Flp-In T-REx HEK293 cell line that stably expresses TetR. TetR repression is relieved by the addition of doxycycline. (**B**) Gene-regulatory activity of MS2-responsive ribozyme switches in human cells. Relative BFP levels are reported for transiently transfected cells harboring the indicated ribozyme switch constructs or controls in the absence and presence of MS2 (1 mg/l doxycycline). (**C**) Gene-regulatory activity of MS2-responsive ribozyme switches to optimized ligand in human cells. Relative BFP levels are reported for transiently transfected constructs encoding ribozyme switch sequences cotransfected with a transfection control plasmid. Reported values are the geometric mean ± SD from biological duplicates and normalized to the non-cleaving control (sTRSVctrl).

We also tested the *in vitro* identified MS2-responsive ribozyme switch designs for gene-regulatory and ligand-responsive activities in a human cell line. A ribozyme switch characterization construct was designed in which the ribozyme switches and non-switch controls were placed in the 3′ UTR of a reporter gene encoding BFP (Figure 5A). A doxycycline-inducible expression cassette for MS2 was located on the same plasmid, in which MS2 expression was under the control of a CMV promoter with two downstream tetracycline operator (TetO) sites (CMV-TetO_2_). All constructs were characterized in a Flp-In T-REx HEK293 cell line, which stably expresses the tetracycline repressor (TetR). Thus, transcription of the protein ligand is inhibited by TetR and the addition of doxycycline to the cell culture media activates transcription of the protein ligand.

Ribozyme switch designs were initially characterized through transient transfection of the characterization construct into the Flp-In T-REx HEK293 cell line and subsequent flow cytometry analysis. The data demonstrate that the different switch designs exhibit different gene-regulatory properties. For example, the transmitter designs (MS2-A) exhibited low to moderate levels of gene knockdown activity (Figure 5B). Three of the designs, MS2-A2, -A3 and -A7, exhibited increases in BFP levels (ranging from 1.3- to 1.8-fold) in response to doxycycline-induced MS2 expression (Figure 5B), with MS2-A2 exhibiting the highest switching activity of all tested designs. Both of the loop-transmitter designs (MS2-B) exhibited gene knockdown activity and achieved both ON and OFF responses to MS2 (Figure 5B), with MS2-B1 exhibiting a 1.4-fold reduction and MS2-B3 exhibiting a 1.6-fold increase in BFP levels in response to MS2 expression. As in the yeast assay (Supplementary Figure S4A), non-switching controls did not exhibit gene knockdown activity or responsiveness to MS2 under identical assay conditions (Supplementary Figure S4B). Taken together, these results indicate that protein-responsive ribozyme switches can exhibit activities in both yeast and mammalian cells.

### An MS2 variant results in improved switch response to ligand

We next examined whether optimization of the protein ligand can improve the ligand sensitivity of the designs that exhibited moderate levels of switching in the initial characterization assay. MS2 binds to its aptamer in the dimerized form (48), and once bound will multimerize to form a capsid (49). We hypothesized that multimerization of the ligand may negatively impact the activity of the ribozyme switches. Thus, we examined the switch response to an alternative version of the MS2 ligand, a fused dimer containing two amino acid substitutions (V75E and A81G) to prevent multimerization (50). As the MS2 monomer must dimerize to bind to the aptamer (48), expressing the protein as a fused dimer roughly doubles the effective ligand concentration.

We assayed a subset of ribozyme switch designs that responded to MS2 in our initial screening experiments (Figure 5B) for sensitivity to the fused dimer MS2 variant using the previously described *in vivo* characterization system (Figure 5A). We performed the characterization assays with the optimized MS2 ligand (2MS2mut) similarly to our initial experiments in the Flp-In T-REx HEK293 cell line. Our results indicate that all examined switches were equally or more responsive to 2MS2mut than to MS2 (Figure 5C). MS2-B3 exhibited the highest increase in BFP levels in response to 2MS2mut (3.6-fold), while MS2-B1 exhibited the greatest decrease (3.8-fold). The observed increase in sensitivity to 2MS2mut is likely due to the lack of multimerization and the increased effective ligand concentration.

Based on the results of these experiments, we explored additional improvements to the switch designs. MS2-A2 differs from MS2-A1 in the sequence of loop I, which we hypothesized may result in the observed increase in gene knockdown and switching activities for MS2-A2. Thus, we modified the designs of MS2-A3 and -A7 by changing the loop I sequence to that of MS2-A2, generating MS2-A10 and -A11, respectively. We assayed these new designs with the 2MS2mut ligand (Figure 5C). The data indicate that MS2-A10 and -A11 exhibited increased gene knockdown and switching activities relative to MS2-A3 and -A7. MS2-A11 exhibited the greatest response to ligand (4.1-fold) of all ON switches tested in the human cell line. MS2-A11 also exhibited improved gene knockdown and switching activities in the yeast host (8.5-fold activation in response to MS2; Figure 4C). These results demonstrate protein-responsive ribozyme switches that exhibit high gene-regulatory activities in response to ligand in a human cell line.

### RNA device gene-regulatory activities in yeast and mammalian hosts correlate with *in vitro* cleavage activities at different Mg^2+^ concentrations

By prototyping the MS2-A and MS2-B devices by their *in vitro* SPR-determined cleavage and binding activities (Figure 3), we identified candidate devices that functioned in yeast (Figure 4) and mammalian (Figure 5) cells. However, the *in vivo* gene-regulatory activities of the devices in yeast and mammalian hosts were not significantly correlated to each other or the SPR-determined cleavage activities (*k*_d_) (Supplementary Figure S5). To examine the cause of this discrepancy, we quantified *in vitro* cleavage kinetics on a subset of MS2-A and MS2-B devices in the absence and presence of MS2 protein through a gel-based cleavage assay.

**Figure 6.**
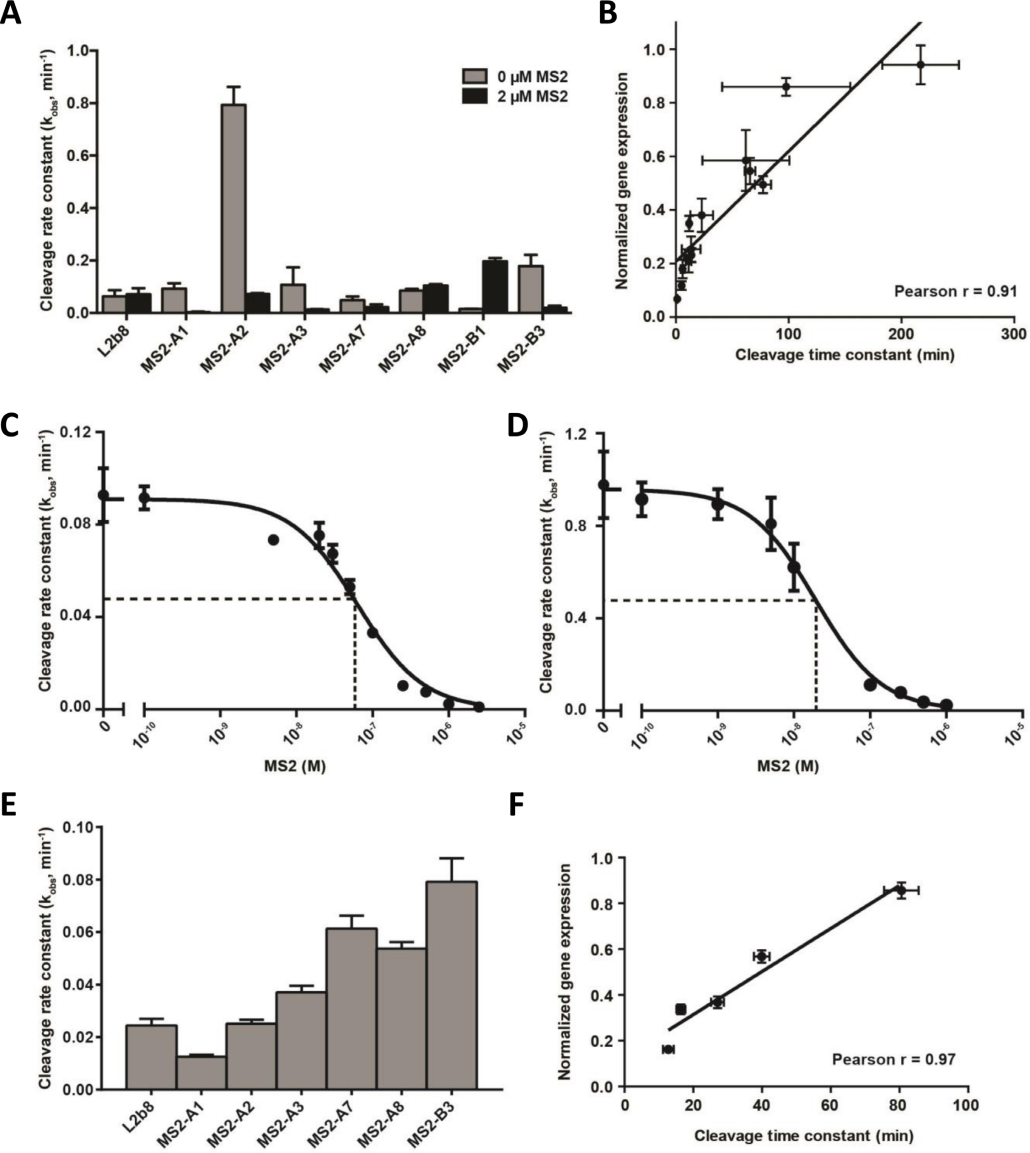
*In vitro* gel-based cleavage activity characterizations of protein-responsive RNA devices. (**A**) Cleavage rate constants for MS2-responsive RNA devices at 500 μM MgCl_2_. Gel-based cleavage assays were performed at 37°C in 500 μM MgCl_2_, 150 mM NaCl, 1 mM DTT and 10 mM HEPES (pH 7.4). Cleavage rate constants (*k*_obs_) are determined by one phase exponential fit (*R*^2^ > 0.99) of the cleavage kinetics of each assay (Supplementary Figure S6). Reported *k*_obs_ are the mean ± SD of at least three independent cleavage assays performed for each device at the indicated ligand concentrations. (**B**) Correlation analysis of RNA device *in vivo* gene-regulatory activities in yeast (Figure 4; ‘No MS2’ and ‘+MS2’ conditions) and *in vitro* cleavage activity (cleavage time constant; *k*_obs_^−1^). Pearson correlation coefficient (*r*): 0.89, slope = 0.006 ± 0.0007. (**C** and **D**) Concentration dependence of cleavage rate constants for (C) MS2-A1 and (D) MS2-A2 RNA devices. Cleavage rate constants (*k*_obs_) were determined at MS2 protein concentrations spanning five orders of magnitude and fit to a three-parameter logistic equation (*R*^2^ > 0.96). Dotted lines indicate IC_50_, the MS2 protein concentration at which the RNA device cleavage rate constant is halfway between maximum and minimum values. Cleavage rate constants reported at each MS2 concentration are the mean ± SD of at least three independent assays. Representative cleavage kinetics are presented in Supplementary Figure S8 and Supplementary Figure S9. (**E**) Cleavage rate constants for MS2-responsive RNA devices at 200 μM MgCl_2_. Gel-based cleavage assays were performed at 37°C in 200 μM MgCl_2_, 150 mM NaCl, 1 mM DTT and 10 mM HEPES (pH 7.4). Cleavage rate constants (*k*_obs_) are reported as the mean ± SD from fit of cleavage kinetics (Supplementary Figure S10) to one phase exponential (*R*^2^ > 0.99) of at least three independent assays for each device. (**F**) Correlation analysis of RNA device *in vivo* basal level gene-regulatory activities in mammalian cells (Figure 5; ‘No MS2’ condition) and *in vitro* cleavage activity (cleavage time constant; *k*_obs_^−1^). Pearson correlation coefficient (*r*): 0.97, slope = 0.008 ± 0.001.

The gel-based cleavage assays were performed on radiolabeled transcripts at physiologically relevant reaction conditions at 37°C in the presence and absence of 2 μM MS2 protein (Figure 6A, Supplementary Figure S6). The cleavage rate constants for the theophylline-responsive control device (L2b8) were comparable in the absence and presence of MS2 protein, indicating that MS2 protein has no non-specific effect on the cleavage activity. In contrast, for almost all the MS2-responsive ON switches, *k*_obs_ values measured in the presence of MS2 protein were lower than those measured in the absence of MS2 protein, indicating that MS2 protein binding to the aptamer shifts the distribution between the two functional conformations to the ribozyme-inactive state, resulting in slower cleavage activity as expected. For the OFF switch (MS2-B1), *k*_obs_ in the presence of MS2 protein is higher, indicating that MS2 protein binding shifts the conformational distribution toward the ribozyme-active state, resulting in faster cleavage activity as expected. The MS2-A8 device cleavage activity (*k*_obs_) slightly increases, from 0.08 to 0.10 min^−1^ in the presence of MS2 protein, supporting the observed OFF-switch behavior in yeast (Figure 4B). We compared the corresponding cleavage time constants (*k*_obs_^−1^) to the yeast gene-regulatory activities (Figure 4B; ‘No MS2’ and ‘+MS2’ condition). The cleavage time constants and *in vivo* yeast gene-regulatory activities exhibit a strong linear correlation (Figure 6B; Pearson product-moment correlation coefficient (Pearson *r*): 0.89). The *in vivo* mammalian gene-regulatory activities (Figure 5C) also correlated to the cleavage time constants, but this correlation was not significant at a *P*-value of 0.01 (Supplementary Figure S7; Pearson *r*: 0.58). These results suggest that the *in vitro* gel-based cleavage assay conditions are more reflective of *in vivo* RNA device cleavage in yeast cells than mammalian cells.

The observed reduction in ribozyme-based device cleavage activity in the presence of MS2 supports the proposed device mechanism of gene regulation. Gel-based cleavage assays were performed on two RNA devices, MS2-A1 and -A2, over a range of MS2 protein concentrations to further characterize the protein ligand-responsiveness of device cleavage activity (Supplementary Figures S8 and S9). As previously observed for allosteric ribozymes, the dependence of *k*_obs_ on MS2 concentration is well characterized by a sigmoidal, three-parameter logistic equation (Figure 6C and D) (28–30). The IC_50_, or MS2 protein concentration at which the cleavage rate constant is half-maximal, is determined as 63 ± 19 nM and 19 ± 4 nM for MS2-A1 and -A2 devices, respectively. These IC_50_ values are ∼2.5× higher, but on the same order of magnitude as the SPR-determined *K*_D_ for MS2-A1 (Figure 3C; 25 ± 6 nM) and -A2 (Figure 6D; 6 ± 3 nM), which supports the use of the *K*_D_ values as a proxy for ligand-responsiveness of device designs.

The results suggest that our *in vitro* cleavage assay conditions are reflective of device cleavage activity in yeast cells, and to a much lesser extent to the cleavage activity in mammalian cells. While there is a multitude of physiological differences between the hosts that may affect RNA device cleavage and/or cleavage-induced gene regulation, we investigated the effect of Mg^2+^ concentration. Mg^2+^ plays an important role in RNA secondary structure formation and tertiary interactions in the HHRz cleavage reaction (40,42,51–52). Researchers have observed the dependence of cleavage activity (*k*_obs_) on Mg^2+^ varies between different HHRz species (53). Thus, it is plausible that the Mg^2+^-dependent *k*_obs_ profile could vary between different RNA device designs. Intracellular, cytosolic free Mg^2+^, in most cell types, is generally estimated between 500 μM and 1 mM (54–59). However, the lower range estimates can be as low as 50 μM (60) and 200 μM (61,62). Thus, it is conceivable that there is significant variation in intracellular Mg^2+^ concentrations between the yeast and mammalian hosts, which leads to differential RNA device cleavage and gene-regulatory patterns.

To investigate the Mg^2+^ dependence on RNA device cleavage activities, we examined *k*_obs_ at a lower physiological Mg^2+^ concentration. Gel-based cleavage assays were performed using the same conditions as above, except at 200 μM MgCl_2_ in the absence of MS2 protein (Figure 6E, Supplementary Figure S10). We find the RNA device rank order is the same in terms of increasing cleavage time constant (at 200 μM MgCl_2_) and *in vivo* mammalian basal gene-regulatory activities (Figure 5C). Furthermore, the cleavage time constants measured at 200 μM MgCl_2_ exhibit a strong linear correlation when compared to *in vivo* mammalian basal gene-regulatory activities (Figure 6F; Pearson *r*: 0.97), but not when compared to those same values for yeast (Supplementary Figure S11; *r*: −0.29). Taken together, our results imply that the *in vivo* gene-regulatory activities in both yeast and mammalian eukaryotic hosts can be predicted by measuring cleavage activities at different, physiologically relevant Mg^2+^ conditions.

### Ribozyme switches respond to protein ligand in both the nucleus and cytoplasm

Understanding the ligand localization requirements of an RNA device is critical to its downstream implementation in regulatory networks. The device may be unresponsive if the ligand of interest is localized to a cellular compartment that is not compatible with its mechanism of action. Protein ligands are often primarily localized to either the nucleus or cytoplasm, such that all of the protein-responsive RNA devices developed to date are limited to responding to ligands localized to one of these two compartments (6,8–11). In the case of a ribozyme ON switch (i.e. ligand binding prevents ribozyme cleavage), if the ribozyme cleaves prior to export from the nucleus and the ligand is present in only the cytoplasm, the switch will be unresponsive to ligand. Alternatively, if the ligand is present in only the nucleus, the ribozyme may cleave after export to the cytoplasm, thereby limiting the switch response to the ligand. We utilized directed localization of the protein ligand to investigate the impact of ligand localization on ribozyme switch activity and shed insight into the mechanism of action of the ribozyme switch, such as when the ribozyme cleaves relative to transcription, nuclear export and translation.

The MS2 (14 kDa) and 2MS2mut (28 kDa) ligands are expected to be present in both the nucleus and the cytoplasm as they are small enough to passively diffuse through the nuclear pore without the aid of any nuclear transport machinery (63). To control localization of the protein ligand, we created 2MS2mut variants with either an N-terminal nuclear localization sequence derived from Simian virus 40 (SV40) (64) (NLS-2MS2mut) or a C-terminal nuclear export sequence derived from protein kinase A inhibitor a (PKIa) (65) (2MS2mut-NES). Western blotting and immunostaining data indicated that although 2MS2mut-NES was effectively localized to the cytoplasm, the NLS-2MS2mut was present in both the cytoplasm and the nucleus (Supplementary Figure S12). We hypothesized that although NLS-2MS2mut was being actively transported into the nucleus, its small size allowed it to passively diffuse out of the nucleus and accumulate in the cytoplasm. To achieve more effective localization of the protein ligand, we created a 2MS2mut-DsRed fusion protein to increase ligand size and modified it with either the N-terminal NLS (NLS-2MS2mut-DsRed) or the C-terminal NES (2MS2mut-DsRed-NES). Expression of the fusion proteins was under the control of a doxycycline-inducible CMV-TetO_2_ promoter and this construct was placed on a plasmid that also encoded constitutive expression of a BFP reporter, which served as an unlocalized reporter protein control. We transiently transfected Flp-In T-REx HEK293 cells with plasmids encoding expression of each of the protein variants and imaged the cells using confocal fluorescence microscopy (Supplementary Figure S13). The data demonstrate that while 2MS2mut-DsRed exhibited the same distribution throughout the cell as BFP, NLS-2MS2mut-DsRed was localized to the nucleus and 2MS2mut-DsRed-NES was localized to the cytoplasm. Thus, the results indicate that the variants of the fusion protein localized to the expected subcellular locations.

**Figure 7.**
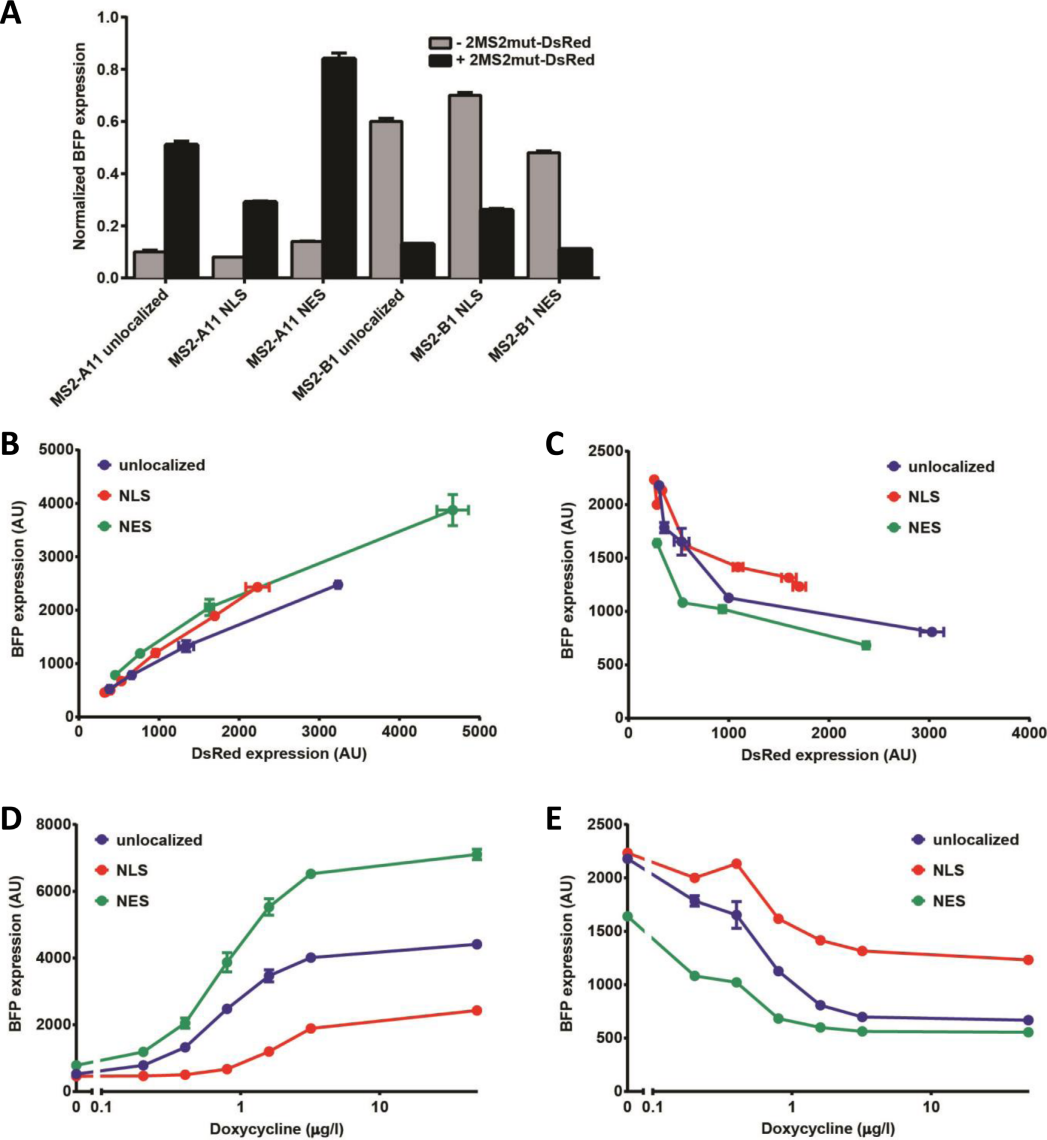
Effect of ligand localization on protein-responsive ribozyme switch activity. (**A**) Gene-regulatory activity of MS2-responsive ribozyme switches in human cells for unlocalized (2MS2mut-DsRed), nuclear localized (NLS-2MS2mut-DsRed) and cytoplasmic localized (2MS2mut-DsRed-NES) ligand. Reported BFP values are the geometric mean ± SD from biological duplicates and normalized to the non-cleaving control (sTRSVctrl). (**B** and **C**) Gene-regulatory activity of (B) MS2-A11 and (C) MS2-B1 for unlocalized, nuclear localized and cytoplasmic localized ligand as a function of doxycycline concentration (0–50 μg/l). Reported BFP values are the geometric mean ± SD from biological duplicates, except MS2-B1 at 0.8 μg/l, unlocalized (singlet). (**D** and **E**) Gene-regulatory activity of (D) MS2-A11 and (E) MS2-B1 for unlocalized, nuclear localized and cytoplasmic localized ligand as a function of ligand concentration. Ligand levels are reported as the DsRed geometric mean ± SD from biological duplicates, except MS2-B1 at 0.8 μg/l, unlocalized (singlet). BFP and DsRed levels are reported for stably integrated constructs encoding the indicated ribozyme switch and ligand expression cassettes at the indicated doxycycline or ligand levels.

We next examined the effects of protein ligand localization on the gene-regulatory activities of the ribozyme switches. The activities of the ON (MS2-A11) and OFF (MS2-B1) switches that exhibited the highest activation ratios were examined with the unlocalized, nuclear-localized and cytoplasmic-localized versions of the ligand in a stable expression assay. The expression constructs were integrated into a Flp-In T-REx HEK293 cell line to generate stable cell lines. Ribozyme switches responded to the nuclear-localized ligand to a lesser extent than to either the unlocalized or cytoplasmic-localized ligands, which result in similar response levels (MS2-A11: NLS, 3.6-fold; unlocalized, 6.4-fold; NES, 6.5-fold; MS2-B1: NLS, 2.8-fold; unlocalized, 4.6-fold; NES, 4.3-fold) (Figure 7A). Both ribozyme switches responded similarly to the 2MS2mut-DsRed ligand as to 2MS2mut ligand (Supplementary Figure S14), indicating that fusion to the fluorescent reporter did not impact switch response. The lower response observed for the nuclear-localized ligand may be explained by either a specific effect of nuclear localization of the ligand on switch response or a reduced steady-state level of the nuclear-localized protein compared to the other variants.

To examine the relationship between ligand expression level and switch response for the three localization variants of 2MS2mut-DsRed, we measured the gene-regulatory activities of both switches in stable cell lines grown across a range of doxycycline concentrations resulting in a range of protein ligand levels (Figure 7B, C, Supplementary Figure S15). The data indicate that MS2-A11 and MS2-B1 exhibited a lower response to the nuclear-localized ligand than to either the unlocalized or cytoplasmic-localized ligand variants at all doxycycline concentrations tested (Figure 7B and C). However, a comparison of gene-regulatory activities at identical concentrations of ligand (i.e. DsRed fluorescence levels) reveals that the three protein variants yielded similar quantitative relationships between switch activities and ligand levels (Figure 7D and E). These results indicate that ribozyme switch response is primarily dependent on protein ligand levels, with ligand localization having only a small effect, such that nuclear and cytoplasmic localization of ligand are each sufficient for achieving a substantial switch response.

## DISCUSSION

We have described a method for generating RNA devices that respond to protein inputs that are located in the nucleus and/or cytoplasm. We further describe an *in vitro* screening strategy for identifying candidates for *in vivo* implementation based on binding and cleavage parameters. Using our strategy, we have developed both ON and OFF ribozyme switches responsive to the MS2 coat protein that exhibit activation ratios as high as 6.5- and 8.5-fold in mammalian and yeast cells, respectively. We observed the *in vivo* gene-regulatory activities of the RNA devices in mammalian and yeast cells to be correlated with *in vitro* cleavage rate activities assayed at different, physiologically relevant Mg^2+^ concentrations, 0.2 and 0.5 mM, respectively.

The RNA device design methodology previously established for small molecule-responsive ribozyme-based devices (18) was utilized in the development of the MS2-A series ON switches (Figure 2A). Our designs integrate the transmitter into loop II of the sTRSV HHRz (Figure 1A), which is predicted to result in a GU wobble base pairing of the second and third nucleotide bases in loop II (Figure 2A) (41). As these bases form important tertiary contacts in the sTRSV HHRz (Figure 1A) (40), we hypothesized that transmitter sequences that destabilize this undesired GU wobble pair would lead to higher cleavage activity and more stringent gene-regulatory control. Analysis of previously developed small molecule-responsive RNA devices with rationally designed (18) and selected transmitter sequences (23) is consistent with this hypothesis. In this study, we observed that the identity of the first base pair of the transmitter sequence immediately following loop II is significant to cleavage activity. For example, MS2-A series devices with a GU/UG wobble pair (MS2-A1, -A2, -A3, -A7, -A8) have significantly greater cleavage activity than devices with a GC Watson–Crick base pair at this position (MS2-A4, -A5, -A6) (Figure 3A). We suspect that in contrast to the Watson–Crick base pair, the wobble pair in the first transmitter position destabilizes undesired base pairings of the second and third HHRz loop II nucleotide bases, retaining important tertiary interactions that ultimately results in higher *in vitro* cleavage and lower *in vivo* gene-regulatory activities.

Our study also describes a novel RNA device transmitter design methodology for creating both ON and OFF switches in mammalian and yeast cells that involve modulating only the HHRz loop structure (MS2-B series). In these device designs, ligand binding results in formation and breaking of base pairs in a HHRz loop for ON and OFF switches, respectively (Figure 2B). One drawback of these transmitter loop-displacing ON switch designs is that they rely on ligand binding modulating a base pairing interaction within the aptamer sequence, which may not be generally extendable to other ligand-aptamer pairs and requires specific information about the ligand-aptamer interaction. However, similar protein-binding aptamers have been characterized (66,67), which should be readily amendable to this loop-displacing transmitter strategy.

Through our *in vitro* SPR-based screening strategy we identified RNA devices that were functional in yeast and mammalian cells. As has been observed with small molecule-responsive RNA devices (23), we found that *in vitro* gel-determined cleavage time constants assayed at 500 μM Mg^2+^ were well correlated with *in vivo* gene-regulatory activities in yeast cells (Figure 6B). The RNA device gene-regulatory activities in mammalian cells did not significantly correlate to *in vitro* cleavage activities assayed at 500 μM Mg^2+^ (Supplementary Figure S7), but did with cleavage activities measured at lower Mg^2+^ concentrations (200 μM; Figure 6F). Our observations suggest a variation in Mg^2+^ concentration between yeast and (HEK293) mammalian cells that is significant for HHRz and ribozyme-based device activities, which have been reported to exhibit different cleavage activity profiles at varying Mg^2+^ levels (53). As such, for the RNA devices tested in both yeast (Figure 4) and mammalian cells (Figure 5), which encompass five different transmitter sequences, we do not find significant correlation between gene-regulatory activities between the two cell types. This finding is in contrast to earlier studies with small molecule-responsive RNA devices, which observed gene-regulatory activities in yeast and mammalian cells to be correlated (15). However, in this earlier study, RNA devices with only three different transmitter sequences were compared. If we remove one of the transmitter sequences from our analysis (i.e. both MS2-A1, A2), we also observe the regulatory activities between yeast and mammalian cells to be correlated. These findings highlight the importance of screening and characterizing *in vitro* device parameters under assay conditions that are reflective of the physiological conditions of the specific cell type desired for *in vivo* deployment.

Our results also demonstrate that the protein-responsive ribozyme switches are a unique class of RNA devices that can respond to ligand in either the nucleus or cytoplasm. This property is somewhat surprising for an ON switch, which cleaves in the absence of ligand. Depending on the relative rates of transcription, mRNA export and ribozyme cleavage, one might expect an ON switch to either cleave during or immediately after transcription if ligand is absent from the nucleus, or to cleave after nuclear export if ligand is absent from the cytoplasm. However, our data suggest that the ribozyme switches do not cleave substantially before nuclear export when ligand is present in only the cytoplasm, nor do they cleave substantially in the cytoplasm when the ligand is present in only the nucleus. A possible explanation for the observed activity with cytoplasmic-localized ligand is that ribozyme cleavage in the nucleus is low, possibly due to prevention of proper HHRz folding due to the binding of proteins that form the messenger ribonucleoprotein, thereby reducing ribozyme cleavage prior to export of the transcript from the nucleus and exposure to the ligand. A possible explanation for the observed activity with nuclear-localized ligand is that the ligand binds the mRNA in the nucleus and remains bound to the mRNA during and after export. The ability of mRNA harboring an MS2 aptamer to carry the ligand out of the nucleus has been previously demonstrated (39). While dissociation of the ligand from the ribozyme switch in the cytoplasm is expected to be favored in this situation, a slow *k*_off_ and conformational switching rate would contribute to providing sufficient time for the mRNA to be translated before cleavage can occur. Future experiments could further examine the mechanism of action of ribozyme switches, by studying the *in vivo* response of switches exhibiting a range of kinetic dissociation rates, cleavage rates and conformational switching rates to localized ligands.

Ribozyme-based devices have become an important class of gene-control elements in synthetic biology, with demonstrated applications ranging from enzyme evolution, to gene therapy, to cellular therapeutics (20–21,68). Our studies provide an important contribution to the growing body of work on ribozyme-based devices by extending the class of ligands these RNA devices can detect to proteins and uncovering the unique capability of these RNA devices to respond to ligand in both the nuclear and cytoplasmic compartments. While we demonstrate switches that respond to MS2 and variant proteins, extension of this platform to diverse protein ligands by leveraging existing RNA aptamer-protein pairs will be an important next step. Such future efforts to continue to develop and leverage this flexible gene-control platform will be important for extension to applications requiring the detection of diverse biomarkers, such as novel diagnostic tools and genetic systems for controlling cellular behavior in response to those biomarkers.

## SUPPLEMENTARY DATA

Supplementary Data are available at NAR Online.

SUPPLEMENTARY DATA
