# Supplementary material for: Protein-responsive ribozyme switches in eukaryotic cells
Source: Nucleic Acids Res. 2014 Oct 1;42(19):12306–21. doi: 10.1093/nar/gku875 (PMC4231745; doi:10.1093/nar/gku875)
Supplement: SUPPLEMENTARY DATA [file supp_42_19_12306__index.html]

Protein-responsive ribozyme switches in eukaryotic cells — Protein-responsive ribozyme switches in eukaryotic cells — SUPPLEMENTARY DATA 

# Protein-responsive ribozyme switches in eukaryotic cells

## SUPPLEMENTARY DATA

**Files in this Data Supplement:**

- SUPPLEMENTARY DATA
