# Supplementary material for: Protein-responsive ribozyme switches in eukaryotic cells
Source: Nucleic Acids Res. 2014 Oct 1;42(19):12306–21. doi: 10.1093/nar/gku875 (PMC4231745; doi:10.1093/nar/gku875)
Supplement: SUPPLEMENTARY DATA [file supp_gku875_nar-01977-r-2014-File009.pdf]

Andrew B. Kennedy, James V. Vowles, Leo d'Espaux, Christina D. Smolke

Protein-responsive ribozyme switches in eukaryotic cells

SUPPLEMENTARY INFORMATION

Supplementary Materials and Methods

Supplementary Figure S1. Plasmid maps for constructs used to characterize RNA devices in yeast and mammalian cells.

Supplementary Figure S2. Schematic overview of SPR-based methods for characterization of RNA device binding and cleavage kinetics.

Supplementary Figure S3. Representative SPR sensorgrams for the MS2-A and MS2-B RNA devices kinetic binding assays with MS2 protein.

Supplementary Figure S4. Gene-regulatory activity of MS2-responsive ribozyme switches and non-cleaving controls in yeast and human cells.

Supplementary Figure S5. Comparison of RNA device *in vivo* gene-regulatory activities in yeast and mammalian cells, and *in vitro* SPR-determined cleavage activity.

Supplementary Figure S6. Representative gel-based cleavage assays for determining cleavage rate constants (k_{obs}) of MS2-A and MS2-B RNA devices generated through a previously-reported cis-blocking and trans-activating strategy.

Supplementary Figure S7. RNA device *in vivo* gene-regulatory activities in mammalian cells compared to *in vitro* cleavage rate constants determined at 500 μM $MgCl_2$.

Supplementary Figure S8. Representative gel-based cleavage assays of the MS2-A1 RNA device dose response of the cleavage rate constant (k_{obs}) to MS2.

Supplementary Figure S9. Representative gel-based cleavage assays of the MS2-A2 RNA device dose response of the cleavage rate constant (k_{obs}) to MS2.

Supplementary Figure S10. MS2-A and MS-B RNA devices cleavage kinetics at low (200 μM) $MgCl_2$.

Supplementary Figure S11. RNA device *in vivo* gene-regulatory activities in yeast cells compared to *in vitro* cleavage rate constants determined at 200 μM $MgCl_2$.

Supplementary Figure S12. Subcellular localization of 2MS2mut ligand with and without localization signals.

Supplementary Figure S13. Subcellular localization of 2MS2mut-DsRed ligand with and without localization signals.

Supplementary Figure S14. Gene-regulatory activity of MS2-responsive ribozyme switches with 2MS2mut with or without fused DsRed.

Supplementary Figure S15. 2MS2mut-DsRed expression levels as a function of doxycycline concentration.

Supplementary Table S1. DNA sequences of all protein-responsive RNA devices and flanking sequences used for *in vitro* cis-blocked constructs and *in vivo* yeast and mammalian expression contexts.

Supplementary Table S2. Free energies (ΔG, kcal/mol) of individual conformations (HHRz-active and -inactive) and the energy difference (ΔG, kcal/mol) between the free energies of these two conformations predicted by RNAstructure5.3.

SUPPLEMENTARY MATERIALS AND METHODS

Cellular fractionation and extraction

Cell culture media were replaced with media containing 1 mg/l doxycycline to derepress the CMV-TetO$_2$ promoter 1–1.5 hr before transfection. Cytoplasmic and nuclear extracts were prepared 50 hr after transfection using the CelLytic NuCLEAR Extraction Kit (Sigma-Aldrich, St. Louis, MO) with isotonic lysis buffer and IGEPAL CA-630 detergent. Modifications were made to the manufacturer's protocol to minimize cross-contamination between the nuclear and cytoplasmic fractions. Briefly, cells were washed with PBS, scraped off of the culture dishes, and centrifuged at 500xg for 5 min. Packed cells were resuspended in isotonic lysis buffer and incubated for 1 min on ice, then incubated with IGEPAL CA-630 at a final concentration of 0.04% on ice for 3 min. Lysed cells were centrifuged at 5000xg for 30 sec and the cytoplasmic fraction (supernatant) was collected. The pelleted nuclei were washed with isotonic lysis buffer and 0.04% IGEPAL CA-630, then centrifuged at 5000xg for 30 sec. The pelleted nuclei were resuspended in extraction buffer and agitated at 4°C for 30 min. Lysed nuclei were centrifuged at 18000xg for 10 min and the nuclear fraction (supernatant) was collected.

Immunoblotting

A standard Bradford assay using Protein Assay reagent (Bio-Rad Laboratories, Hercules, CA) was performed on cytoplasmic and nuclear extracts with a BSA standard to determine protein concentrations. Samples containing approximately 20–30 μg of protein were run on NuPAGE 4–12% Bis-Tris Gels (Life Technologies, Carlsbad, CA) in NuPAGE MOPS buffer (Life Technologies) at 150 V for 1 hr. Transfer was performed with extra thick blot paper (Bio-Rad Laboratories) and 0.45 μm Protran nitrocellulose transfer membrane (Whatman, Little Chalfont, England) in 2x NuPAGE transfer buffer (Life Technologies) and 20% methanol using a Trans-Blot SD Semi-Dry Transfer Cell (Bio-Rad Laboratories) at 15 V for 15 min. Membranes were blocked with TBST (20 mM Tris, 137 mM sodium chloride, 0.1% Tween-20) and 5% bovine serum albumin (BSA) (EMD Millipore, Billerica, MA) at room temperature for 1 hr, then rinsed with TBST twice for 5 min. Membranes were probed with rabbit anti-enterobacteriophage MS2 coat protein, anti-GAPDH, and anti-HDAC1 polyclonal antibodies (EMD Millipore) in TBST and 1% BSA at 4°C for 16 hr, then rinsed with TBST twice for 5 min. Membranes were probed with sheep anti-rabbit IgG, horseradish peroxidase (HRP) conjugate polyclonal antibody (EMD Millipore, Billerica, MA) at room temperature for 1 hr, then rinsed with TBST twice for 5 min. HRP signal was detected using SuperSignal West Pico Chemiluminescent Substrate (Thermo Scientific, Waltham, MA) according to the manufacturer's instructions and a G:BoxChemi XT4 imaging system (Syngene, Cambridge, England). Band intensity was calculated with GeneTools software (Syngene).

Immunofluorescence microscopy

Flp-In T-REx HEK293 cells with stably integrated 2MS2mut constructs were seeded at $1x10^5$ cells/ml in 1 ml (4-chambered coverglass) DMEM with 10% FBS, 100 mg/l hygromycin B, and 1 mg/l doxycycline to derepress the CMV-TetO$_2$ promoter. Approximately 43 hr after seeding cells were washed with phosphate buffered saline (PBS) (Life Technologies) and fixed for 15–20 min using HistoChoice MB tissue fixative (AMRESCO, Solon, OH). Cells were washed twice with PBS, blocked with PBS and 1.5% BSA for 1 hr, and washed with PBS for 5 min. Cells were probed with rabbit anti-enterobacteriophage MS2 coat protein at 4°C for approximately 17 hr, then washed with PBS for 5 min. Cells were probed with sheep anti-rabbit fluorescein conjugated [F(ab')$_2$ fragments] polyclonal antibody (Chemicon International, Temecula, CA) for 30 min, then washed three times with PBS for 5 min. Cell nuclei were counterstained using 250 µg/l 7-AAD (Life Technologies) in PBS for 5 min, then washed with PBS. Cells were imaged on a Zeiss Axiovert 200M fluorescence microscope (Carl Zeiss, Oberkochen, Germany) with a 20x objective using the AxioVision software (Zeiss). Images were exported and brightness and contrast were adjusted using FIJI.

Confocal fluorescence microscopy

Approximately 30 min after transfection with plasmids encoding 2MS2mut-DsRed, 1 mg/l doxycycline was added to derepress the CMV-TetO$_2$ promoter, and 23 hr later media were replaced with media containing 1 mg/l doxycycline. Cell nuclei were counterstained using 250 nM SYTO 16 (Life Technologies) 24 hr after addition of media containing doxycycline. Cells were imaged 24 hr after counterstaining on a Leica TCS SP8 confocal microscope (Leica Microsystems, Wetzlar, Germany) with a 20x objective using the Leica Application Suite Advanced Fluorescence software (Leica Microsystems). Images were exported and brightness and contrast were adjusted using FIJI.

A

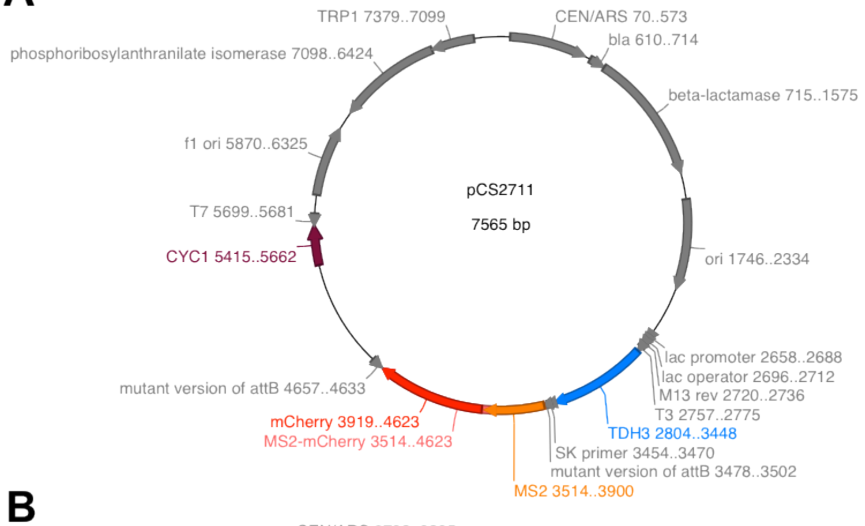
pCS2711
7565 bp
TRP1 7379..7099
CEN/ARS 70..573
bla 610..714
phosphoribosylanthranilate isomerase 7098..6424
beta-lactamase 715..1575
f1 ori 5870..6325
T7 5699..5681
CYC1 5415..5662
ori 1746..2334
lac promoter 2658..2688
lac operator 2696..2712
M13 rev 2720..2736
T3 2757..2775
mutant version of attB 4657..4633
TDH3 2804..3448
mCherry 3919..4623
MS2-mCherry 3514..4623
SK primer 3454..3470
mutant version of attB 3478..3502
MS2 3514..3900

B

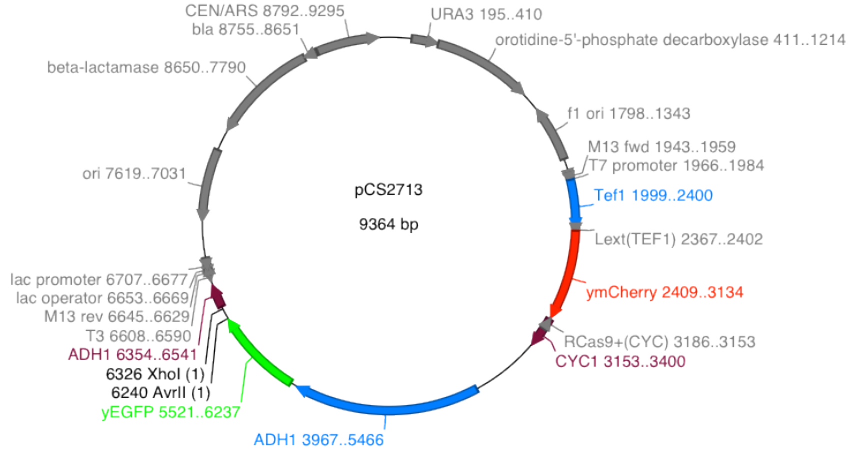
pCS2713
9364 bp
CEN/ARS 8792..9295
bla 8755..8651
URA3 195..410
orotidine-5'-phosphate decarboxylase 411..1214
beta-lactamase 8650..7790
f1 ori 1798..1343
M13 fwd 1943..1959
T7 promoter 1966..1984
ori 7619..7031
Tef1 1999..2400
Lext(TEF1) 2367..2402
lac promoter 6707..6677
lac operator 6653..6669
ymCherry 2409..3134
M13 rev 6645..6629
T3 6608..6590
RCas9+(CYC) 3186..3153
ADH1 6354..6541
CYC1 3153..3400
6326 XhoI (1)
6240 AvrII (1)
yEGFP 5521..6237
ADH1 3967..5466

C

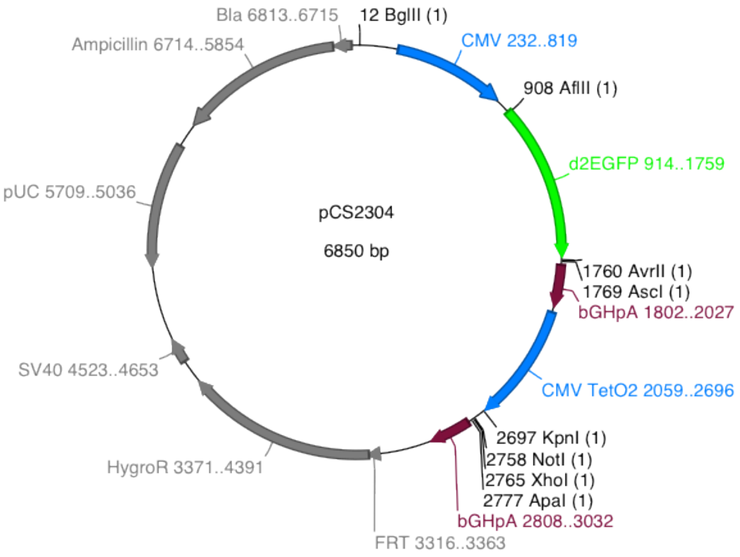

D

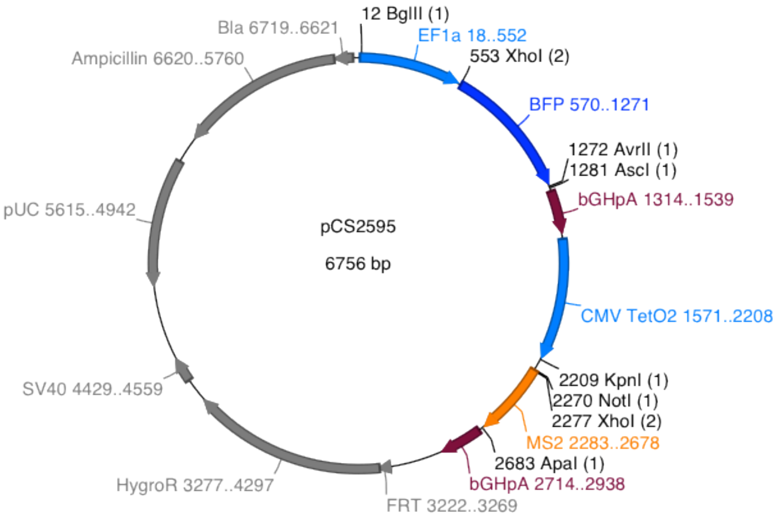

Supplementary Figure S1. Plasmid maps for constructs used to characterize RNA devices in yeast and mammalian cells. (**A**) pCS2711, (**B**) pCS2713 (derived from pCS1748), (**C**) pCS2304, (**D**) pCS2595.

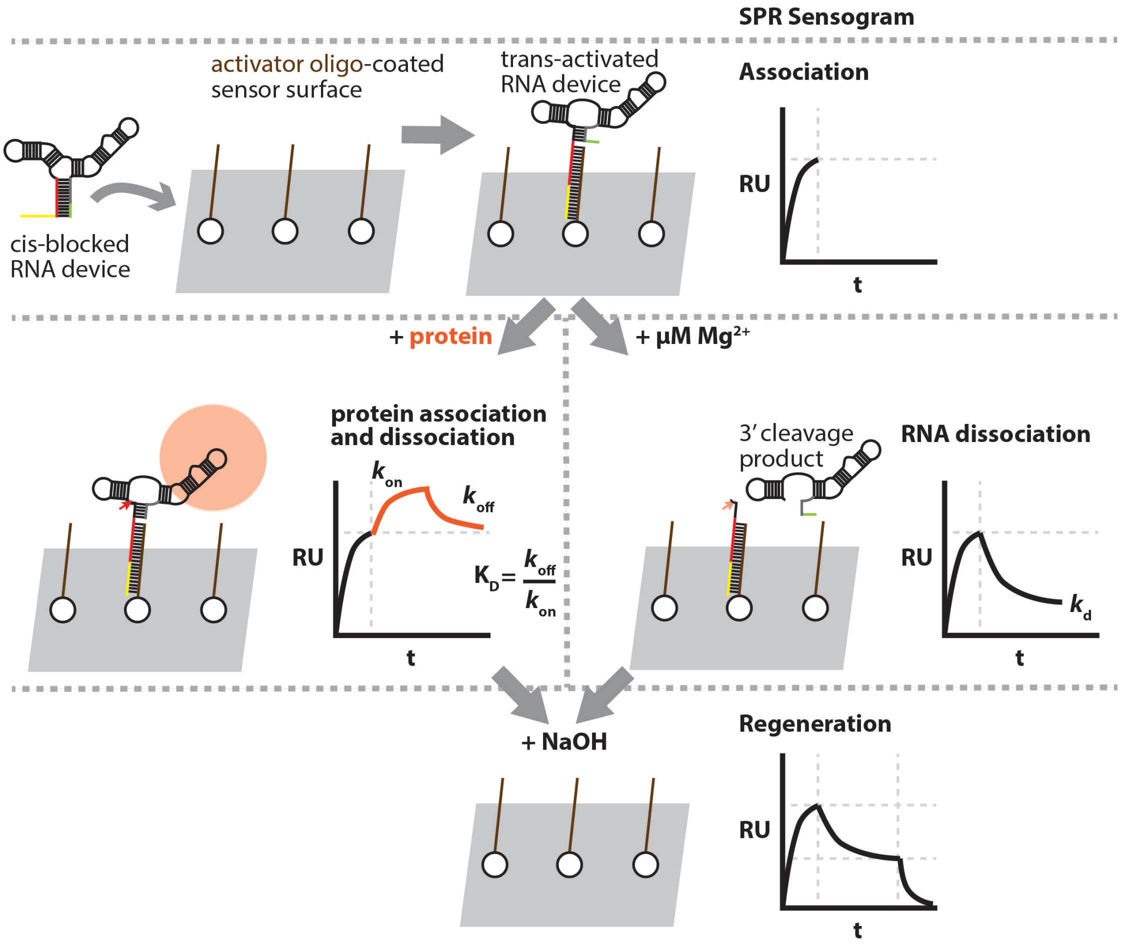

Supplementary Figure S2. Schematic overview of SPR-based methods for characterization of RNA device binding and cleavage kinetics. Cis-blocked constructs of the RNA devices containing a toehold (yellow line) and blocking (red line) sequences are transcribed *in vitro* and hybridized to an activator DNA (brown line) immobilized on the SPR surface, allowing the RNA device to fold correctly (for full description of methodology see (13). The protein binding kinetics and affinity (left), and RNA dissociation rate constants (right) are measured in separate assays as described in the Methods section.

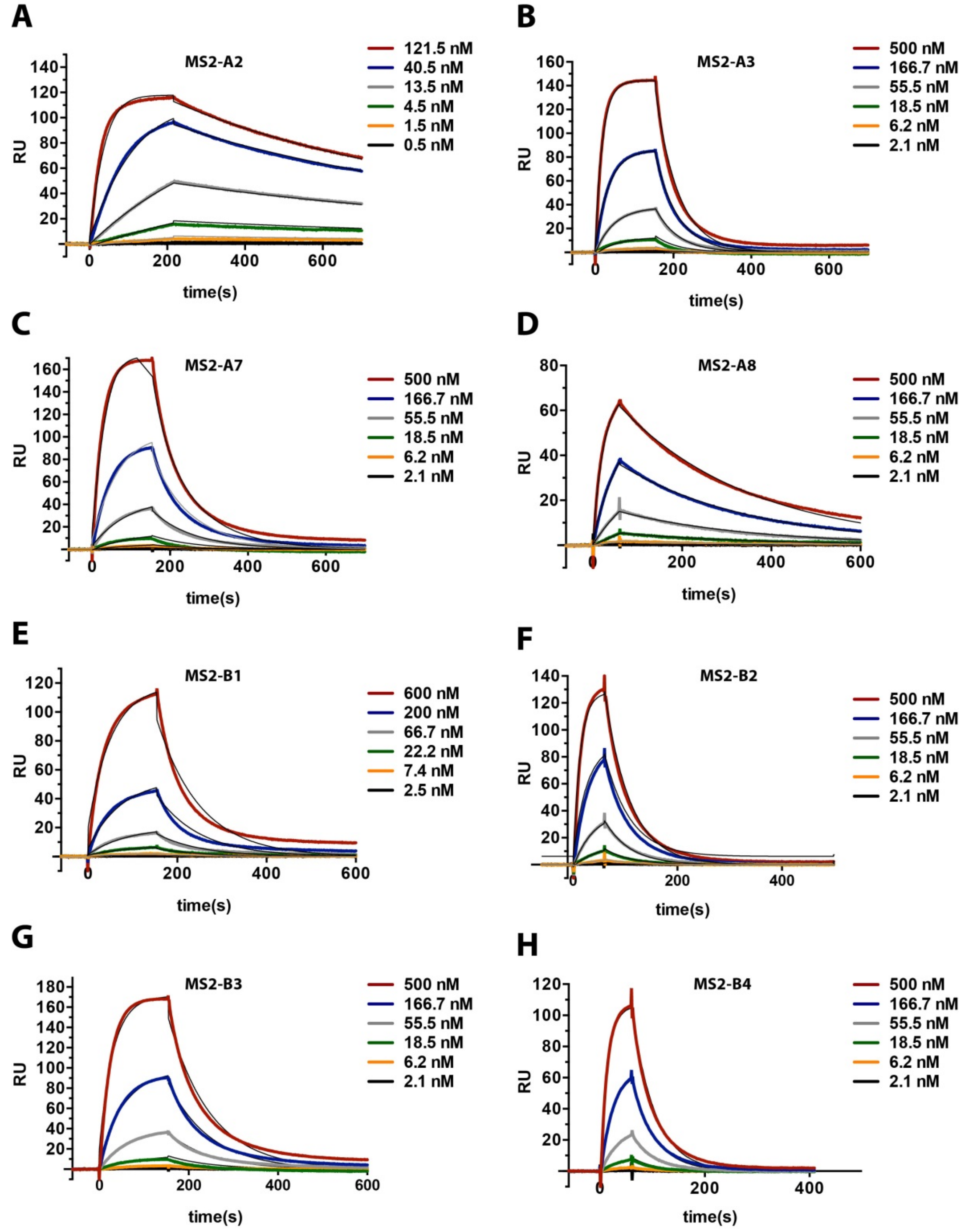

I

RNA Device	k_{on} x 10^{-4} ($M^{-1}s^{-1}$)	k_{off} x 10^{2} (s^{-1})	K_D (nM)
MS2-A1	8.2 ± 3.0	0.19 ± 0.03	24.5 ± 5.7
MS2-A2	25.5 ± 10.6	0.14 ± 0.02	6.08 ± 2.4

MS2-A3	14.0 ± 6.0	3.9 ± 1.9	253 ± 13
MS2-A7	6.1 ± 3.0	1.6 ± 0.7	279 ± 26
MS2-A8	7.5 ± 2.0	0.36 ± 0.02	50 ± 16
MS2-B1	1.9 ± 0.7	1.12 ± 0.10	630 ± 152
MS2-B2	18.4 ± 7.8	4.12 ± 1.18	237 ± 45
MS2-B3	4.7 ± 0.3	1.39 ± 0.30	296 ± 53
MS2-B4	21.9 ± 9.7	4.57 ± 0.89	224 ± 51

Supplementary Figure S3. Representative SPR sensorgrams for the MS2-A and MS2-B RNA devices kinetic binding assays with MS2 protein. (**A**) MS2-A2, (**B**) MS2-A3, (**C**) MS2-A7, (**D**) MS2-A8, (**E**) MS2-B1, (**F**) MS2-B2, (**G**) MS2-B3, (**H**) MS2-B4 representative plots. Overlaid in each plot are the association and dissociation portion of the sensograms from binding MS2 protein at six different concentrations. Replicate runs used in parameter fitting are not shown. SPR data is double referenced and fit to 1:1 kinetic binding model to calculate the protein-RNA dissociation constant, K_D, from the ratio k_{on} and k_{off} kinetic rate constants. (**I**) Kinetic and affinity constants for MS2 protein interaction of the selected MS2-A and MS2-B RNA devices that passed the SPR-cleavage screening assay. Reported values are mean ± SD of at least three independent assays.

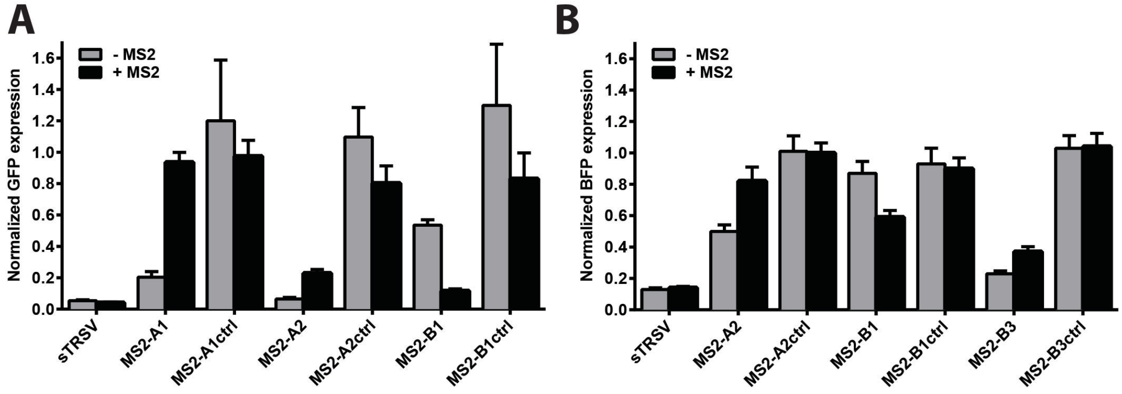

Supplementary Figure S4. Gene-regulatory activity of MS2-responsive ribozyme switches and non-cleaving controls in (**A**) yeast and (**B**) human cells. (**A**) Relative GFP levels are reported for cells harboring the indicated ribozyme switch constructs or controls in the absence and presence of MS2. Reported values are the geometric mean ± SD from biological triplicates and normalized to the non-cleaving control (sTRSVctrl). (**B**) Relative BFP levels are reported for transiently transfected cells harboring the indicated ribozyme switch constructs or controls in the absence and presence of MS2 (1 mg/l doxycycline). Reported values are the geometric mean ± SD from biological duplicates and normalized to the non-cleaving control (sTRSVctrl). All non-cleaving control devices are identical to cleaving devices except for a one base-pair mutation in the catalytic core that abolishes cleavage.

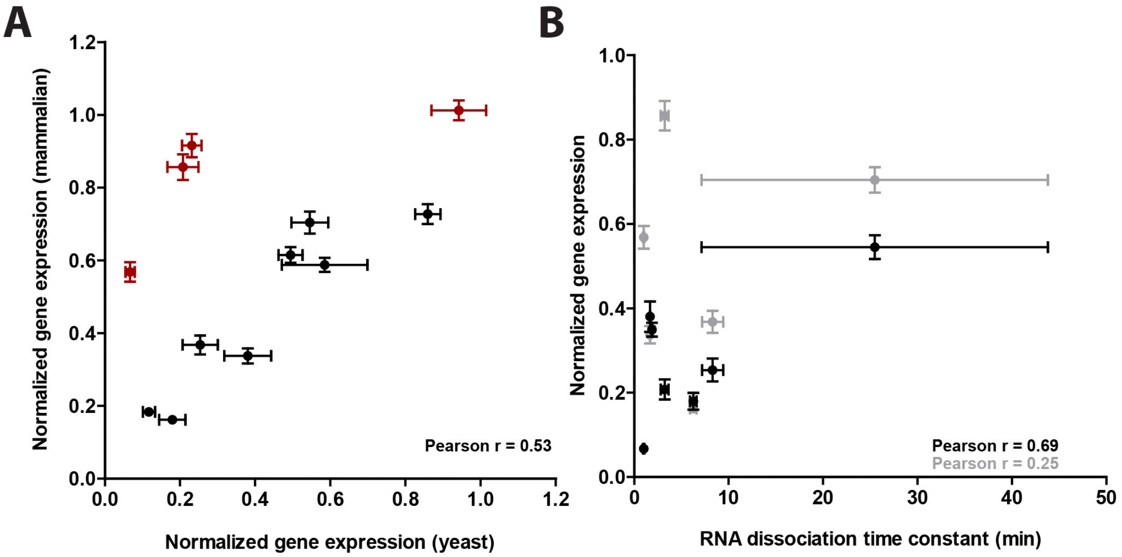

Supplementary Figure S5. Comparison of RNA device *in vivo* gene-regulatory activities in yeast and mammalian cells, and *in vitro* SPR-determined cleavage activity. (**A**) Correlation analysis of *in vivo* gene-regulatory activities of RNA devices in different eukaryotic cell types. RNA device gene-regulatory activities ('No MS2' and '+ MS2' conditions) in yeast cells (Figure 4B) and mammalian cells (Figure 5C) are not significantly correlated, Pearson correlation coefficient (r): 0.53. The correlation becomes significant (r: 0.93) when two RNA devices (red; MS2-A1 and -A2 'No MS2' and '+ MS2' condition) are excluded from the analysis. (**B**) Correlation analysis of RNA device yeast (black; Figure 4B) and mammalian (grey; Figure 5C) *in vivo* gene-regulatory activities and *in vitro* SPR-determined cleavage activity (RNA dissociation time constant (k_d^{-1}); Figure 3B). The relationship between both *in vivo* gene-regulatory activities and SPR-determined *in vitro* cleavage activity was observed not to be significant, with Pearson correlation coefficients (r): 0.69, 0.25 with yeast and mammalian normalized gene expression, respectively.

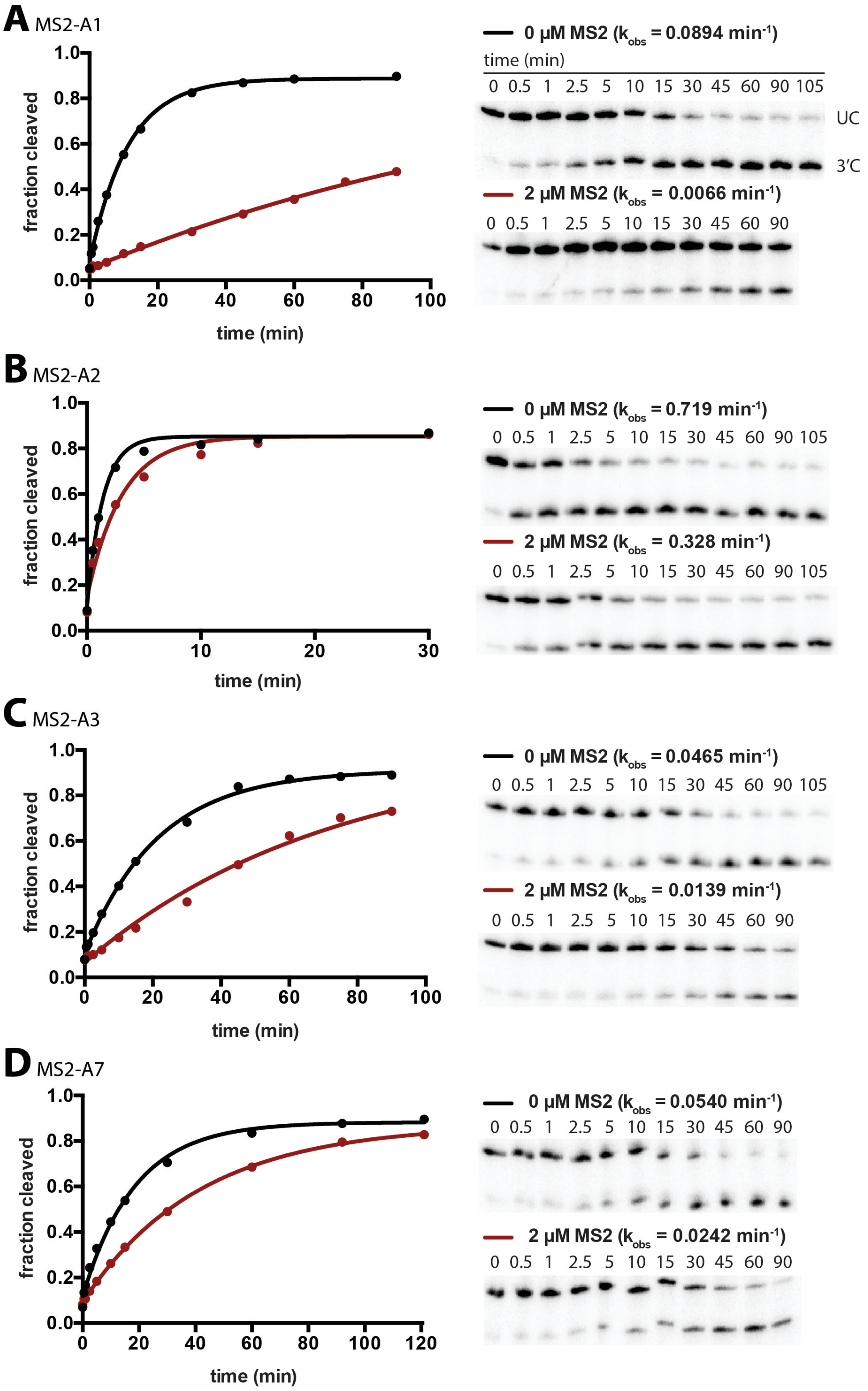
A MS2-A1
fraction cleaved
time (min)
0 μM MS2 (kobs = 0.0894 min-1)
time (min)
0 0.5 1 2.5 5 10 15 30 45 60 90 105
UC
3'C
2 μM MS2 (kobs = 0.0066 min-1)
0 0.5 1 2.5 5 10 15 30 45 60 90
B MS2-A2
fraction cleaved
time (min)
0 μM MS2 (kobs = 0.719 min-1)
0 0.5 1 2.5 5 10 15 30 45 60 90 105
2 μM MS2 (kobs = 0.328 min-1)
0 0.5 1 2.5 5 10 15 30 45 60 90 105
C MS2-A3
fraction cleaved
time (min)
0 μM MS2 (kobs = 0.0465 min-1)
0 0.5 1 2.5 5 10 15 30 45 60 90 105
2 μM MS2 (kobs = 0.0139 min-1)
0 0.5 1 2.5 5 10 15 30 45 60 90
D MS2-A7
fraction cleaved
time (min)
0 μM MS2 (kobs = 0.0540 min-1)
0 0.5 1 2.5 5 10 15 30 45 60 90
2 μM MS2 (kobs = 0.0242 min-1)
0 0.5 1 2.5 5 10 15 30 45 60 90

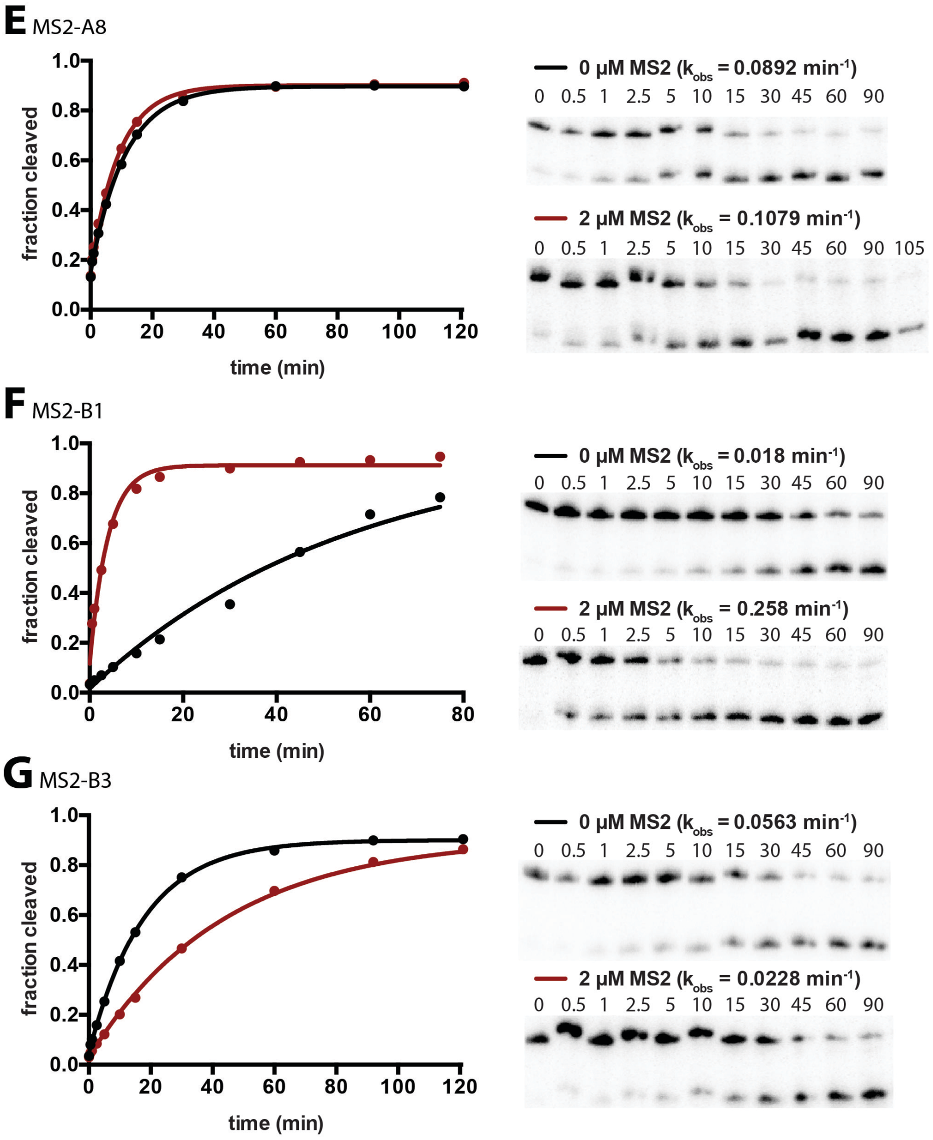
E MS2-A8
fraction cleaved
time (min)
0 μM MS2 (k obs = 0.0892 min-1)
0 0.5 1 2.5 5 10 15 30 45 60 90
2 μM MS2 (k obs = 0.1079 min-1)
0 0.5 1 2.5 5 10 15 30 45 60 90 105
F MS2-B1
fraction cleaved
time (min)
0 μM MS2 (k obs = 0.018 min-1)
0 0.5 1 2.5 5 10 15 30 45 60 90
2 μM MS2 (k obs = 0.258 min-1)
0 0.5 1 2.5 5 10 15 30 45 60 90
G MS2-B3
fraction cleaved
time (min)
0 μM MS2 (k obs = 0.0563 min-1)
0 0.5 1 2.5 5 10 15 30 45 60 90
2 μM MS2 (k obs = 0.0228 min-1)
0 0.5 1 2.5 5 10 15 30 45 60 90

H	Cleavage rate constant (k_{obs}, min^{-1})	
**RNA Device**	**0 µM MS2**	**2 µM MS2**
MS2-A1	0.093 ± 0.020	0.003 ± 0.002
MS2-A2	0.793 ± 0.069	0.073 ± 0.002
MS2-A3	0.108 ± 0.066	0.013 ± 0.001
MS2-A7	0.049 ± 0.014	0.023 ± 0.010
MS2-A8	0.085 ± 0.006	0.104 ± 0.005
MS2-B1	0.015 ± 0.001	0.197 ± 0.012
MS2-B3	0.179 ± 0.043	0.020 ± 0.008

Supplementary Figure S6. Representative gel-based cleavage assays for determining cleavage rate constants (k_{obs}) of MS2-A and MS2-B RNA devices generated through a previously-reported cis-blocking and trans-activating strategy (13). Representative cleavage assays for each construct in absence and presence of 2 µM MS2 protein are shown as: (**A**) MS2-A1, (**B**) MS2-A2, (**C**) MS2-A3, (**D**) MS2-A7, (**E**) MS2-A8, (**F**) MS2-B1, (**G**) MS2-B3 RNA devices. Bands for the full-length uncleaved substrate (UC) and longer cleaved product (3'C) at each time point are shown, the shorter 5'C product is omitted for clarity. Cis-blocked RNA was incubated with the DNA activator in a buffer (150 mM NaCl, 10 mM HEPES (pH 7.4)) for 2 min to activate the blocked RNA. A zero time point aliquot was removed prior to initiating the reaction with addition of $MgCl_2$ to a final concentration of 500 µM. Reactions were quenched; at least seven time points were taken in each cleavage assay to capture the cleavage dynamics of RNA devices exhibiting different cleavage kinetics. Phosphorimaging analysis of relative levels of the UC, 5'C, and 3'C bands was used to determine the fraction cleaved at each time point (F_t). The fraction cleaved at the beginning (F_0) and end of reaction (F_∞) varied between assays, but all assays were well-fit to the single exponential equation (R^2>0.95):

$$F_t=F_0+(F_\infty-F_0)\times(1-e^{-k_{obs}t})$$

The black and red lines represent assays performed at 0 and 2 µM MS2 coat protein, respectively. (**H**) Reported cleavage rate constants (k_{obs}) for each RNA device in the main text are the mean ± SD of at least three independent assays.

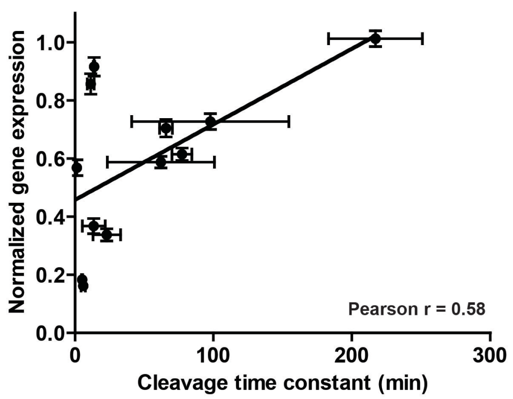

Supplementary Figure S7. RNA device *in vivo* gene-regulatory activities in mammalian cells compared to *in vitro* cleavage rate constants determined at 500 µM $MgCl_2$. Correlation between *in vivo* activities in 'No MS2' and '+MS2 condition' (Figure 5C) to *in vitro* cleavage time constants (at 500 µM $MgCl_2$) in the absence and presence of 2 µM MS2 protein (Figure 6A) is significant, with Pearson correlation coefficient, r: 0.58. Best-fit line of linear regression is shown, with slope 0.003 ± 0.001.

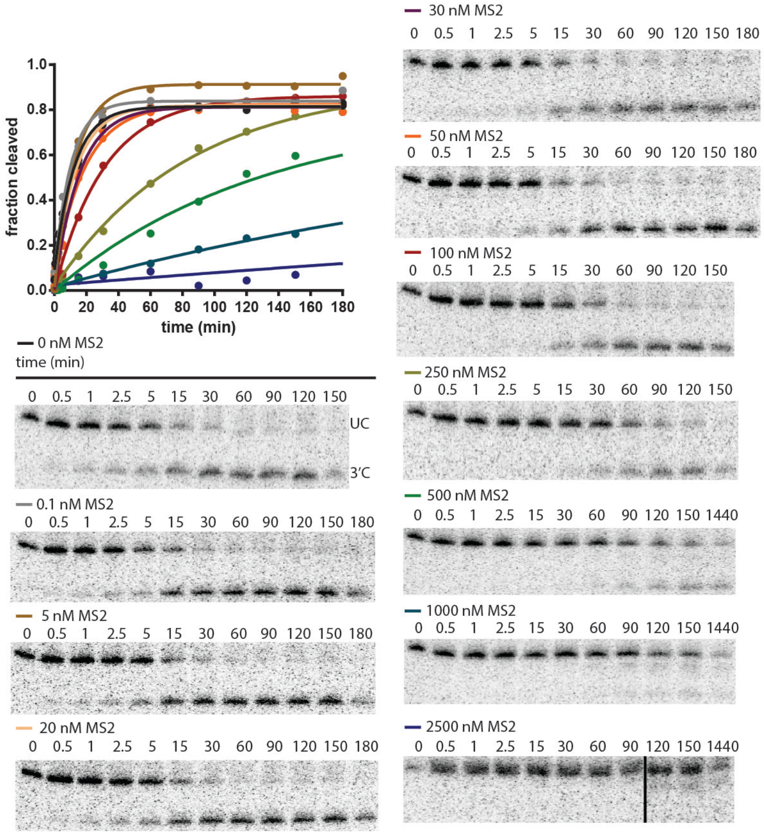

Supplementary Figure S8. Representative gel-based cleavage assays of the MS2-A1 RNA device dose response of the cleavage rate constant (k_{obs}) to MS2. Vertical black line indicates where samples were run on separate gels. Assay conditions and cleavage rate constant determination are as described in Supplementary Figure S6.

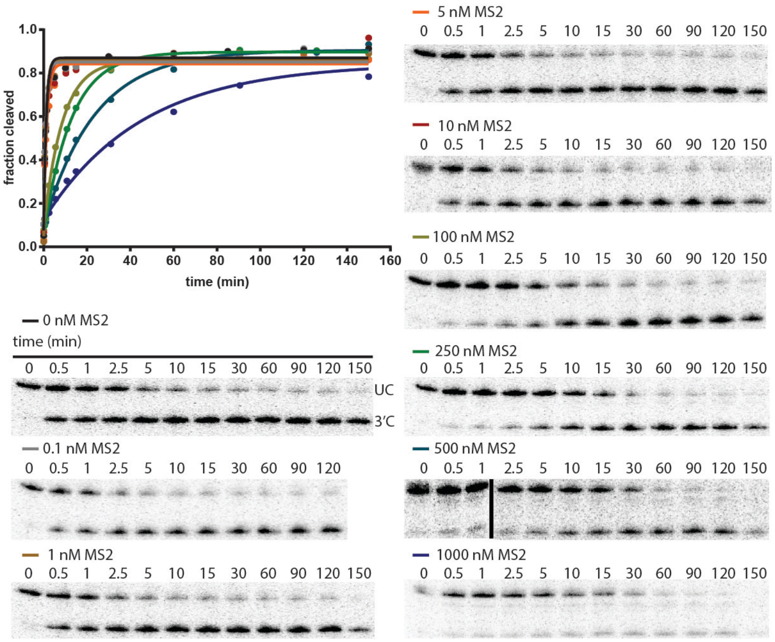

Supplementary Figure S9. Representative gel-based cleavage assays of the MS2-A2 RNA device dose response of the cleavage rate constant (k_{obs}) to MS2. Vertical black line indicates where samples were run on separate gels. Assay conditions and cleavage rate constant determination are as described in Supplementary Figure S6.

A

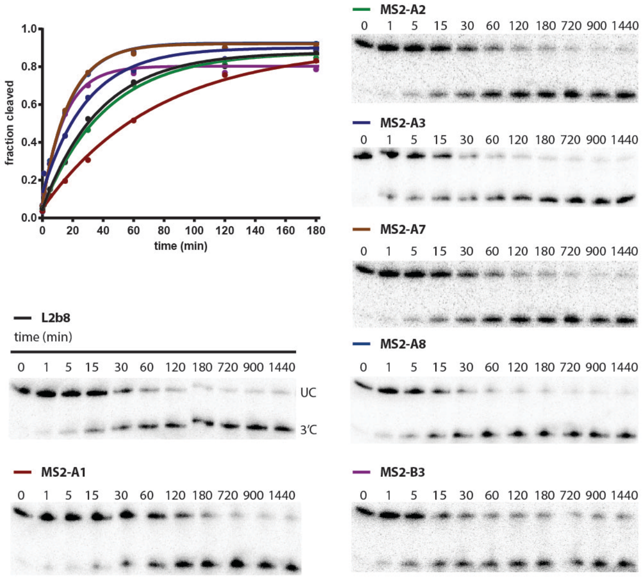

B

RNA Device	Cleavage rate constant (k_{obs}, min^{-1})
L2b8	0.024 ± 0.003
MS2-A1	0.012 ± 0.001
MS2-A2	0.025 ± 0.001
MS2-A3	0.037 ± 0.002
MS2-A7	0.061 ± 0.005
MS2-A8	0.054 ± 0.003
MS2-B3	0.079 ± 0.008

Supplementary Figure S10. MS2-A and MS-B RNA devices cleavage kinetics at low (200 µM) $MgCl_2$. (**A**) Representative gel-based cleavage assays for determining cleavage rate constants (k_{obs}) of L2b8, MS2-A1, MS2-A2, MS2-A3, MS2-A7, MS2-A8, and MS2-B3. Assay conditions are the same as described in Supplementary Figure S6 except $MgCl_2$ concentration is 200 µM. (**B**) Cleavage rate constants determined from fit to one phase exponential (R^2>0.99), mean ± SD are reported from at least three independent assays.

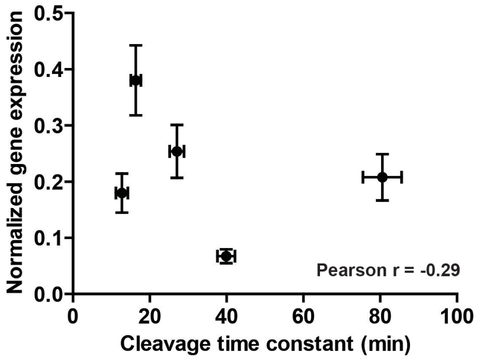

Supplementary Figure S11. RNA device *in vivo* gene-regulatory activities in yeast cells compared to *in vitro* cleavage rate constants determined at 200 μM $MgCl_2$. Correlation between *in vivo* activities in 'No MS2' (Figure 4B) to *in vitro* cleavage time constants (at 200 μM $MgCl_2$) in the absence of MS2 protein (Figure 6E) is not significant, with Pearson correlation coefficient, r: -0.29.

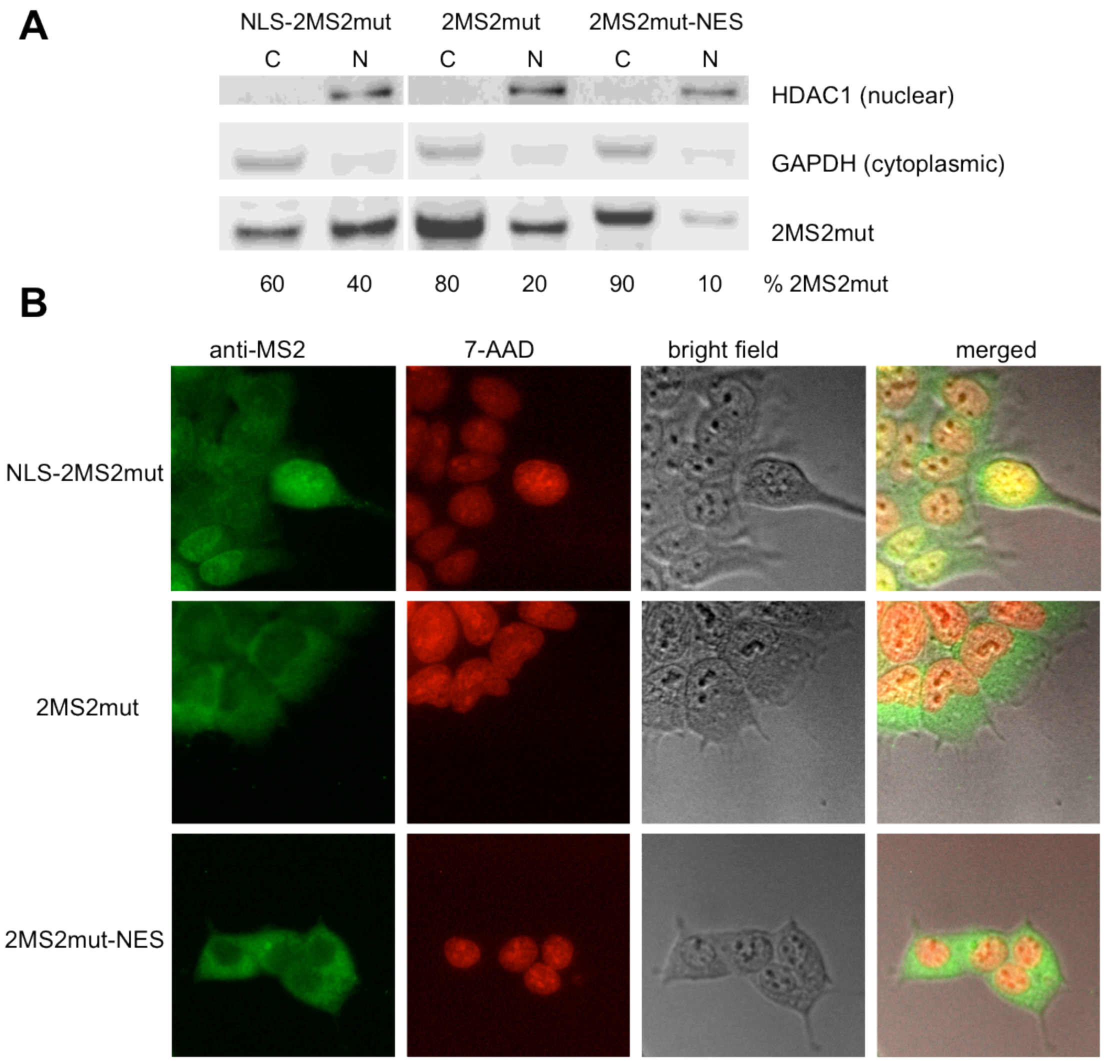

Supplementary Figure S12. Subcellular localization of 2MS2mut ligand with and without localization signals. (**A**) Representative immunoblot image showing subcellular compartment distribution of 2MS2mut, NLS-2MS2mut, and 2MS2mut-NES with nuclear (HDAC1) and cytoplasmic (GAPDH) controls. Percentages are calculated by normalizing quantified band intensity to the number of cells harvested. C, cytoplasm; N, nucleus; HDAC1, histone deacetylase 1; GAPDH, glyceraldehyde 3-phosphate dehydrogenase. (**B**) Representative immunofluorescence microscopy images of HEK293 cells expressing the 2MS2mut, NLS-2MS2mut, and 2MS2mut-NES ligands using the same anti-MS2 antibody as in (**A**). Green, anti-MS2 antibody and fluorescent secondary antibody; red, 7-aminoactinomycin D (7-AAD) nuclear stain.

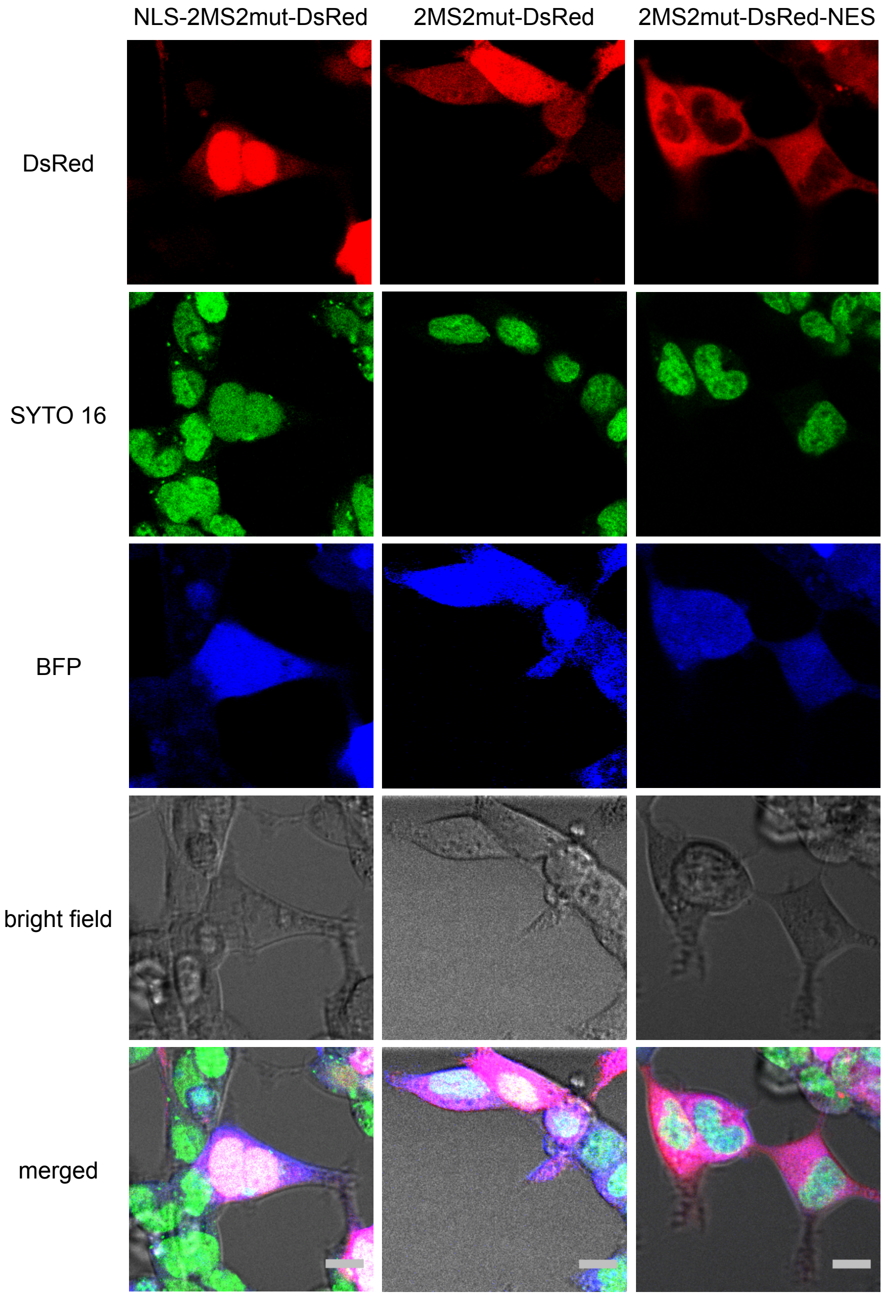

Supplementary Figure S13. Subcellular localization of 2MS2mut-DsRed ligand with and without localization signals. Representative confocal fluorescence microscopy images are shown of HEK293 cells expressing the 2MS2mut-DsRed, NLS-2MS2mut-DsRed, and 2MS2mut-NES-DsRed ligands. Red, DsRed; blue, BFP; green, SYTO 16 nuclear stain. Scale bars are 10 μm.

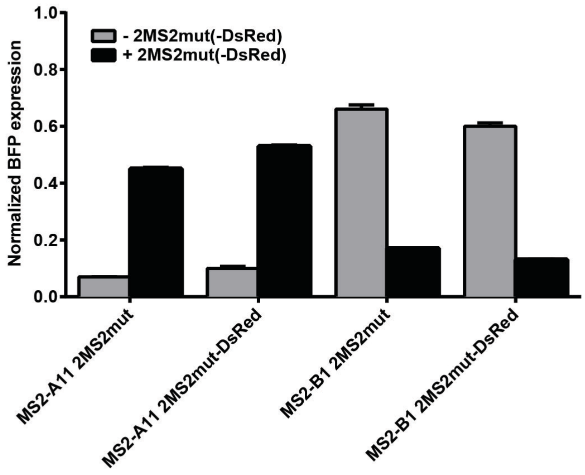

Supplementary Figure S14. Gene-regulatory activity of MS2-responsive ribozyme switches with 2MS2mut with or without fused DsRed. Relative BFP levels are reported for stably integrated constructs encoding the indicated ribozyme switch and ligand expression cassettes in the absence and presence of MS2 (1 mg/l or 50 µg/l (MS2-B11 only) doxycycline). Reported values are the geometric mean ± SD from biological duplicates and normalized to the non-cleaving control (sTRSVctrl).

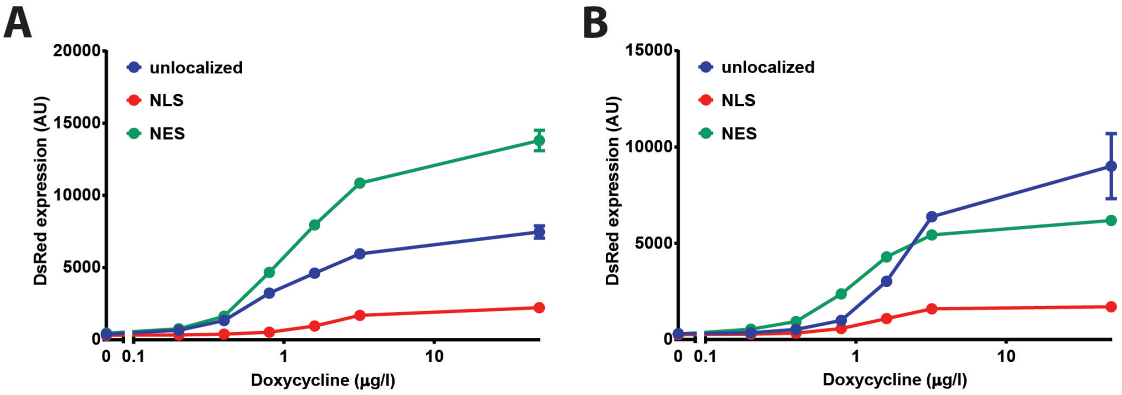

Supplementary Figure S15. 2MS2mut-DsRed expression levels as a function of doxycycline concentration. DsRed levels are reported for stably integrated constructs encoding the indicated ribozyme switch ((**A**) MS2-11, (**B**) MS2-B1) and ligand expression cassettes at the indicated doxycycline levels (0–50 µg/l). Reported values are geometric mean ± SD from biological duplicates, except MS2-B1 at 0.8 µg/l, unlocalized (singlet).

Supplementary Table S1. DNA sequences of all protein-responsive RNA devices and flanking sequences used for *in vitro* cis-blocked constructs and *in vivo* yeast and mammalian expression contexts. For *in vitro* transcription and subsequent cleavage assays, the T7 promoter sequence and activator DNA have been previously described (13).

RNA device name	Ligand	DNA sequence
sTRSV	N/A	GCTGTCACCGGATGTGCTTTCCGGTCTGATGAGTCCGTGAGGACGAAACAGC
sTRSVctrl	N/A	GCTGTCACCGGATGTGCTTTCCGGTCTGATGAGTCCGTGAGGACAAAACAGC
L2b8	theophylline	GCTGTCACCGGATGTGCTTTCCGGTCTGATGAGTCCGTTGTCCATACCAGCATCGTCTTGATGCCCTTGGCAGGGACGGGACGGAGGACGAAACAGC
MS2-A1	MS2 protein	GCTGTCACCGGATGTGCTTTCCGGTCTGATGAGTCCGTTGTCCAGGATCACCGGACGGGACGGAGGACGAAACAGC
MS2-A1ctrl	MS2 protein	GCTGTCACCGGATGTGCTTTCCGGTCTGATGAGTCCGTTGTCCAGGATCACCGGACGGGACGGAGGACAAAACAGC
MS2-A2	MS2 protein	GCTGTCACCGGAATCAAGGTCCGGTCTGATGAGTCCGTTGTCCACCATCAGGGGACGGGACGGAGGACGAAACAGC
MS2-A2ctrl	MS2 protein	GCTGTCACCGGAATCAAGGTCCGGTCTGATGAGTCCGTTGTCCACCATCAGGGGACGGGACGGAGGACAAAACAGC
MS2-A3	MS2 protein	GCTGTCACCGGATGTGCTTTCCGGTCTGATGAGTCCGTGGTCCACCATCAGGGGACTGGACTGAGGACGAAACAGC
MS2-A4	MS2 protein	GCTGTCACCGGATGTGCTTTCCGGTCTGATGAGTCCGTCGTCCAGGATCACCGGACGGGACGGAGGACGAAACAGC
MS2-A5	MS2 protein	GCTGTCACCGGATGTGCTTTCCGGTCTGATGAGTCCGTCGTCCTAGGATCACCAGGACGGGACGGAGGACGAAACAGC
MS2-A6	MS2 protein	GCTGTCACCGGATGTGCTTTCCGGTCTGATGAGTCCGTCGTCCTAGGATCACCAGGAAGGGACGGAGGACGAAACAGC
MS2-A7	MS2 protein	GCTGTCACCGGATGTGCTTTCCGGTCTGATGAGTCCGTTGTCCTAGGATCACCAGGAAGGGACGGAGGACGAAACAGC
MS2-A8	MS2 protein	GCTGTCACCGGATGTGCTTTCCGGTCTGATGAGTCCGTTGCGTAGGATCACCACGTGGCGCGGAGGACGAAACAGC

MS2-A9	MS2 protein	GCTGTCACCGGATGTGCTTTCCGGTCTGATGAGTCC GTTGTAGGATCACCACACGGAGGACGAAACAGC
MS2-A10	MS2 protein	GCTGTCACCGGAATCAAGGTCCGGTCTGATGAGTCC GTGGTCCACCATCAGGGGACTGGACTGAGGACGAA ACAGC
MS2-A11	MS2 protein	GCTGTCACCGGAATCAAGGTCCGGTCTGATGAGTCC GTTGTCCTAGGATCACCAGGAAGGGACGGAGGACG AAACAGC
MS2-B1	MS2 protein	GCTGTCACCGGATGTGCTGCAGGATCACCGCATTTC CGGTCTGATGAGTCCGTGAGGACGAAACAGC
MS2-B1ctrl	MS2 protein	GCTGTCACCGGATGTGCTGCAGGATCACCGCATTTC CGGTCTGATGAGTCCGTGAGGACAAAACAGC
MS2-B2	MS2 protein	GCTGTCACCGGATGTGGTTTCCGGTCTGATGAGTCC GACCATCAGGAGGACGAAACAGC
MS2-B3	MS2 protein	GCTGTCACCGGAATCAAGGTCCGGTCTGATGAGTCC GACCATCAGGAGGACGAAACAGC
MS2-B3ctrl	MS2 protein	GCTGTCACCGGAATCAAGGTCCGGTCTGATGAGTCC GACCATCAGGAGGACAAAACAGC
MS2-B4	MS2 protein	GCTGTCACCGGATTCGGGATCCGGTCTGATGAGTCC GACCATCAGGAGGACGAAACAGC
**Device flanking regions**	**Experiment**	**Sequence**
5'spacer	*in vivo* mammalian	AATAAATAAAA
3'spacer	*in vivo* mammalian	CAAATAAACAAACACTC
5'spacer	*in vivo* yeast	AAACAAACAAA
3'spacer	*in vivo* yeast	AAAAAGAAAAATAAAAA
5'cis-blocking	*in vitro* cleavage	GGGAAACAAACAAAGTTGTTTT
stabilizing	*in vitro* cleavage	TTTGTT

Supplementary Table S2. Free energies (ΔG, kcal/mol) of individual conformations (HHRz-active and -inactive) and the energy difference (ΔG, kcal/mol) between the free energies of these two conformations predicted by RNAstructure5.3 (38).

**RNA device**	**aptamer unformed**		**aptamer formed**		**HHRz→ inactive (aptamer formed)**	**inactive→ HHRz+aptamer formed**
	**HHRz-active**	**HHRz-inactive**	**HHRz-inactive**	**HHRz-active**		
sTRSV	-19.3	N/A	N/A	N/A	N/A	N/A
L2b8	-36.9	N/A	-34.8	N/A	2.1	N/A
MS2-A1	-27.7	N/A	-26.9	N/A	0.8	N/A
MS2-A2	-28.7	N/A	-27.3	N/A	1.4	N/A
MS2-A3	-30.6	N/A	-29.1	N/A	1.5	N/A
MS2-A4	-29.7	N/A	-28.5	N/A	1.2	N/A
MS2-A5	-30.4	N/A	-30.3	N/A	0.1	N/A
MS2-A6	-30.4	N/A	-27.9	N/A	2.5	N/A
MS2-A7	-28.5	N/A	-26.1	N/A	2.4	N/A
MS2-A8	-27.4	N/A	-25.5	N/A	1.9	N/A
MS2-A9	-22.7	N/A	-21.6	N/A	1.1	N/A
MS2-A10	-29.7	N/A	-28.2	N/A	1.5	N/A
MS2-A11	-27.5	N/A	-25.2	N/A	2.3	N/A
MS2-B1	N/A	-24.6	N/A	-22.3	N/A	2.3
MS2-B2	-20.6	N/A	N/A	N/A	N/A	N/A
MS2-B3	-19.7	N/A	N/A	N/A	N/A	N/A
MS2-B4	-21.9	N/A	N/A	N/A	N/A	N/A
